# Hypoxia induces alterations in tRNA modifications involved in translational control

**DOI:** 10.1186/s12915-023-01537-x

**Published:** 2023-02-21

**Authors:** Huanping Guo, Lin Xia, Wei Wang, Wei Xu, Xipeng Shen, Xiao Wu, Tong He, Xuelin Jiang, Yinying Xu, Pan Zhao, Dongmei Tan, Xi Zhang, Yunfang Zhang

**Affiliations:** 1 Medical Center of Hematology, Xinqiao Hospital, State Key Laboratory of Trauma, Burn and Combined Injury, Army Medical University, Chongqing, 400037 China; 2 Chongqing Medical University, Chongqing, 400016 China; 3 Jinfeng Laboratory, Chongqing, 401329 China; 4 Clinical and Translational Research Center of Shanghai First Maternity and Infant Hospital, Shanghai Key Laboratory of Signaling and Disease Research, Frontier Science Center for Stem Cell Research, School of Life Sciences and Technology, Tongji University, Shanghai, 200092 China

**Keywords:** tRNA, tsRNA, RNA modifications, Hypobaric hypoxia

## Abstract

**Background:**

Adaptation to high-altitude hypobaric hypoxia has been shown to require a set of physiological traits enabled by an associated set of genetic modifications, as well as transcriptome regulation. These lead to both lifetime adaptation of individuals to hypoxia at high altitudes and generational evolution of populations as seen for instance in those of Tibet. Additionally, RNA modifications, which are sensitive to environmental exposure, have been shown to play pivotal biological roles in maintaining the physiological functions of organs. However, the dynamic RNA modification landscape and related molecular mechanisms in mouse tissues under hypobaric hypoxia exposure remain to be fully understood. Here, we explore the tissue-specific distribution pattern of multiple RNA modifications across mouse tissues.

**Results:**

By applying an LC-MS/MS-dependent RNA modification detection platform, we identified the distribution of multiple RNA modifications in total RNA, tRNA-enriched fragments, and 17–50-nt sncRNAs across mouse tissues; these patterns were associated with the expression levels of RNA modification modifiers in different tissues. Moreover, the tissue-specific abundance of RNA modifications was sensitively altered across different RNA groups in a simulated high-altitude (over 5500 m) hypobaric hypoxia mouse model with the activation of the hypoxia response in mouse peripheral blood and multiple tissues. RNase digestion experiments revealed that the alteration of RNA modification abundance under hypoxia exposure impacted the molecular stability of tissue total tRNA-enriched fragments and isolated individual tRNAs, such as tRNA^Ala^, tRNA^val^, tRNA^Glu^, and tRNA^Leu^. In vitro transfection experiments showed that the transfection of testis total tRNA-enriched fragments from the hypoxia group into GC-2spd cells attenuated the cell proliferation rate and led to a reduction in overall nascent protein synthesis in cells.

**Conclusions:**

Our results reveal that the abundance of RNA modifications for different classes of RNAs under physiological conditions is tissue-specific and responds to hypobaric hypoxia exposure in a tissue-specific manner. Mechanistically, the dysregulation of tRNA modifications under hypobaric hypoxia attenuated the cell proliferation rate, facilitated the sensitivity of tRNA to RNases, and led to a reduction in overall nascent protein synthesis, suggesting an active role of tRNA epitranscriptome alteration in the adaptive response to environmental hypoxia exposure.

**Supplementary Information:**

The online version contains supplementary material available at 10.1186/s12915-023-01537-x.

## Background

A high-altitude, hypobaric hypoxia environment has been shown deleterious effects on the physical health of the organisms [1], especially for travelers from plain ascending into plateaus (>3500 m), leading to acute mountain sickness (AMS), high-altitude cerebral edema, or high-altitude pulmonary edema due to the lack of oxygen [2–4]. However, the high-altitude populations, such as those in Tibet, exhibit a distinctive suite of physiological traits to respond to extreme hypoxia [5], indicating a systematic regulatory mechanism that exists for high-altitude adaptation. With the wide application of whole-genome sequencing and high-throughput RNA sequencing, genetic evidence for and transcriptome regulation for high-altitude adaptation have been identified [6]. Previous studies revealed that the missense mutations on the *EGLN1* gene [7, 8] and SNPs on the *EPAS1* gene [9] in Tibetan populations might partially explain the generational evolution of adapting to high-altitude environmental exposure. However, whether the epigenetic regulation process is involved in high-altitude hypoxia adaptation is still under investigation. Recently, DNA methylation of the promoter regions of hypoxia-related genes, LINE- 1 [10], and chromatin accessibility [11] were reported to be associated with adaptation to hypoxia during lifetime high-altitude exposure, providing insight into epigenetic regulation in high-altitude adaptation.

RNA modifications have been shown to play pivotal biological roles in maintaining the normal physiological function of organs [12–14]. N^6^-Methyladenosine (m^6^A) is the most enriched internal mRNA modification and has been extensively investigated by researchers worldwide [15]. Reversible m^6^A is dynamically regulated by METTL3 and METTL14 (the writers) and FTO and ALKBH5 (the erasers) and involved in translation and RNA metabolism with its reader proteins [16]. In addition to m^6^A, over 170 types of posttranscriptional RNA modifications have been identified, while only a few of them have been comprehensively investigated [17]. Eukaryotic ribosome RNA (rRNA) and transfer RNA (tRNA) are the most extensively modified cellular RNAs and have been shown to have multifaceted cellular functions that are closely dependent on their RNA modifications [18–20]. For rRNAs, rRNA modifications affect ribosome maturation and protein synthesis in numerous ways [21, 22]. For tRNAs, tRNA modifications contribute to maintaining tRNA clover-leaf-shaped structures and biological functions in translation [23–25]. Moreover, dysregulation of tRNA modifications affects tRNA intracellular localization [26], amino acid charging efficiency [27], codon decoding fidelity [28], tRNA structure stability [29–31], and the generation of various types of tRNA-derived small noncoding RNAs (tsRNAs) (also called tDRs or tRNA-derived fragments, tRFs) [24], which in turn play a pivotal role in cellular transcriptional and translational control in response to various cell stresses [32]. In addition to rRNA and tRNA, RNA modifications on mRNA (such as m^6^A, N^1^-methyladenosine (m^1^A), 5-methylcytosine (m^5^C), and pseudouridine (ψ)) are sensitive to environmental exposure and nutrition supplement alterations [12] and are actively involved in human diseases [17]. However, the dynamic RNA modification regulation landscape on tissue RNA under high-altitude hypoxia exposures is still unknown.

Recently, some studies have linked RNA modification alteration with hypoxic environmental exposure. Metge et al. found that hypoxia exposure induced a different 2′-O-Me methylation pattern on ribosomal RNA to generate ribosomal heterogeneity for hypoxia adaptation [33]. Zhang et al. and others reported that m^6^A modification, together with its writer, eraser, and reader proteins, is involved in hypoxia adaptation in heart disease and cancer [34]. By applying a simulated high-altitude hypobaric oxygen chamber (>5500 m), our previous study also revealed that hypobaric hypoxia exposure could reshape the overall RNA modification landscape for mouse testis and sperm RNAs [35]. In this study, we comprehensively characterized the RNA modification signature of total RNAs, tRNA-enriched fragments, and 17–50-nt sncRNAs across mouse tissues and described the alteration signature of RNA modifications with tissues specificity in response to environmental hypobaric hypoxia exposure. We also identified that the dysregulation of hypoxia-sensitive RNA modifications could destabilize tRNA to RNase and accelerate the biogenesis of tsRNA, which might further slowdown the overall cellular translation process and attenuate cell proliferation to adapt to a hypoxic environment.

## Results

### Identification and characterization of the tissue-specific RNA modification signature in mouse tissues

**Fig. 1:**
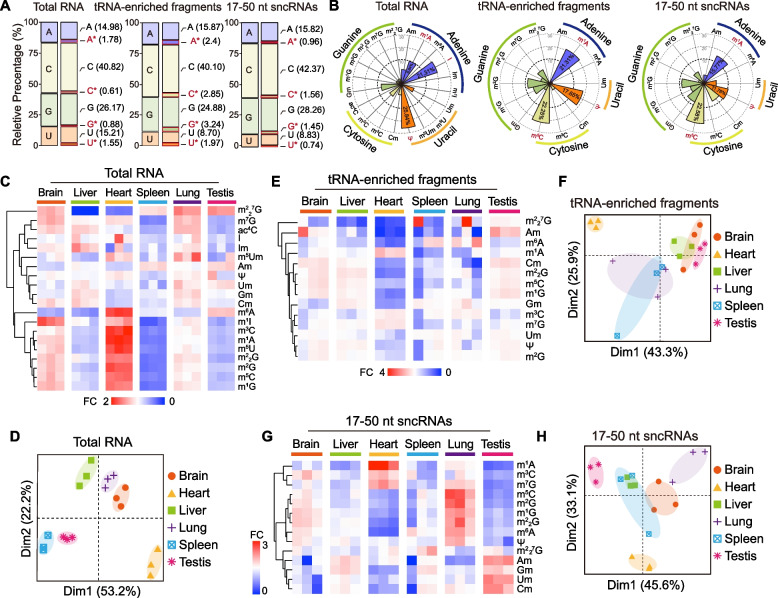
RNA modification landscapes in mouse multiple tissues. **A** Relative percentage of modified and unmodified four nucleotides (adenine, uracil, cytosine, and guanine) in mouse total RNA, tRNA-enriched fragments, and 17–50-nt sncRNAs. The sum of all modified and unmodified nucleotides abundance was considered as 100 and the percentage was calculated by the average of each nuclear base across six tissues (*n* = 3, Additional file 10). **B** The relative proportion of detected RNA modifications across six tissues, the sum of all RNA modifications was considered as 100 and the percentage was calculated by the average of each modification across six tissues. **C**, **E**, **G** The heatmaps showed the relative expression levels of each RNA modification to average across six mouse tissues in total RNA, tRNA-enriched fragments, and 17–50-nt sncRNAs. **D**, **F**, **H** Principal component analysis (PCA) of RNA modifications in total RNA, tRNA-enriched fragments, and 17–50-nt sncRNAs across six mouse tissues

Since RNA modifications have been shown to be pivotal in epitranscriptome regulation [12], it is urgent to illustrate the expression landscape of various RNA modifications across tissues. By using our previously established RNA modification detection platform based on liquid chromatography-tandem mass spectroscopy (LC-MS/MS) [36], we systematically quantified various RNA modification levels for different classes of RNA (total RNAs (rRNAs account for over 80% [37]), poly (A)-enriched RNAs (mainly mRNAs, named mRNA below), ~80-nt RNA fragments (mainly tRNAs, named tRNA-enriched fragments below), and 17–50-nt RNA fragments (named as 17–50-nt sncRNAs below)) across six main tissues in mice (brain, liver, heart, spleen, lung, and testis) (Additional file 1: Fig. S1A). In our RNA modification detection platform, 26 types of nucleobase standards were applied (Additional file 1: Fig. S1B), and 20, 15, 14, and 14 types of RNA modification were successfully quantitated in tissue total RNAs, mRNA, tRNA-enriched fragments, and 17–50-nt sncRNAs, respectively (Fig. 1A, B and Additional file 1: Fig. S1C-D). The data showed that the modified ratios of adenine (A), uracil (U), cytosine (C), and guanine (G) varied among total RNAs, mRNAs, tRNA-enriched fragments, and 17–50-nt sncRNAs (Fig. 1A and Additional file 1: Fig. S1C). Consisted with a previous report [25], tRNA was the most extensively modified class of RNAs, in which the modified nucleotides accounted for 10.46% of the overall nucleotides (Fig. 1A). Among all detected modified nucleotides in mouse total RNAs, Ψ (25.84%) and inosine (I) (21.31%) accounted for the highest percentage, while the 5,2′-O-dimethyluridine (m^5^Um) (0.17%), N1-methylinosine (m^1^I) (0.18%), N2, N2, N7-Trimethylguanosine (m^2^_2_^7^G) (0.22%), and 2′-O-methylinosine (Im) (0.27%) accounted for only a small portion of the modified nucleotides (Fig. 1B). I (42.89%), 2′-O-methylcytidine (Cm) (10.32%) and 2′-O-methylguanosine (Gm) (9.05%) shared more than half of the quantified RNA modifications in mRNAs (Additional file 1: Fig. S1D), whereas m^5^C, m^1^A, and Ψ composed over 50% of detected RNA modifications in tRNA-enriched fragments and 17–50-nt sncRNAs (Fig. 1B).

Due to the relatively low levels of overall RNA modifications on mRNAs (Additional file 1: Fig. S1C), we next focused on analyzing the RNA modification landscape of total RNA, tRNA-enriched fragments, and 17–50-nt sncRNAs across mouse tissues (Fig. 1C–H). The cluster analysis showed that the relative expression levels of different RNA modifications in total RNA, tRNA-enriched fragments, and 17–50-nt sncRNAs were differentially expressed across mouse tissues (Fig. 1C, E, G), and the RNA modification signature in the heart showed the most distinct feature from all other tissues, while the liver and spleen were similar to each other (Fig. 1C, E, G), indicating that the various RNA modifications might play a pivotal role in regulating tissue-specific functions. To further analyze the tissue specificity of RNA modification levels, we performed principal component analysis to classify different tissues with different RNA modification levels. The abundance of multiple RNA modifications in mouse tissues revealed a tissue-specific RNA modification signature that could classify mouse tissues based on RNA modification identity (Fig. 1D, F, H). Compared with total RNA, the mRNAs (Additional file 1: Fig. S1E-F), tRNA-enriched fragments, and 17–50-nt sncRNA showed a moderate classification of different tissues with different RNA modification levels (Fig. 1D, F, H), suggesting that a coordination between different RNA classes was needed to compose the tissue-specific RNA modification identity.

### The tissue-specific RNA modification pattern was associated with the levels of RNA modification enzymes

**Fig. 2:**
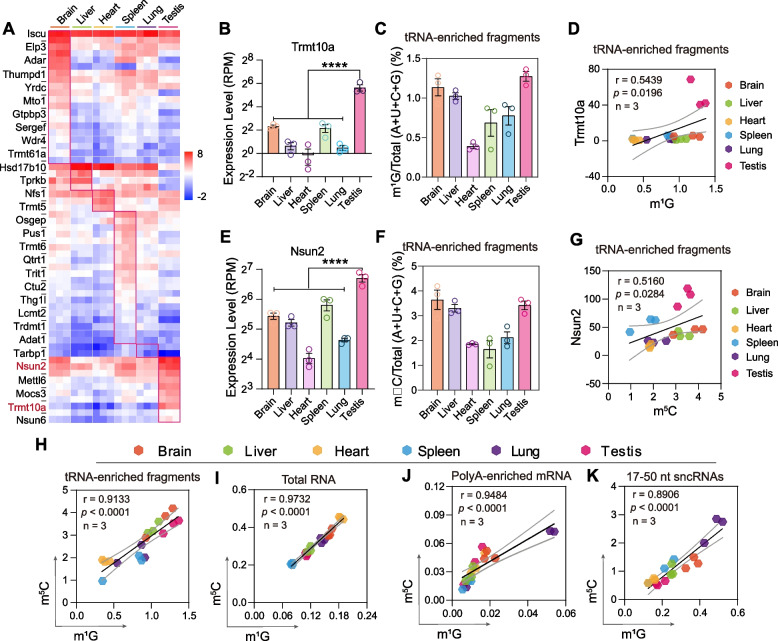
Expression pattern of RNA modification enzymes in mouse tissues. **A** The heatmap shows the expression levels of RNA modification enzymes in six mouse tissues (*n* = 3). **B** The expression level of tRNA methyltransferase 10 homologue A (Trmt10a) obtained by transcriptome sequencing. **C** The relative expression level of m^1^G in tRNA-enriched fragments (*n* = 3, Additional file 10). **D** The linear correlation of Trmt10a and m^1^G in tRNA-enriched fragments. **E** The expression level of tRNA methyltransferase Nsun2 obtained by transcriptome sequencing. **F** The relative expression level of m^5^C in tRNA-enriched fragments. **G** The linear correlation of Nsun2 and m^5^C in tRNA-enriched fragments. **H**–**K** The linear correlation of m^1^G and m^5^C in total RNA, mRNA, tRNA-enriched fragments, and 17–50-nt sncRNAs. Pearson correlation coefficients were computed with GraphPad Prism 8. Statistical analysis in **B** and **D** was conducted using one-way ANOVA with uncorrected Fisher’s LSD

RNA modification levels are dynamically regulated by RNA enzymes, including methyltransferase, demethylases, and RNA modification-specific binding proteins [16]. We next wanted to determine whether the tissue-specific RNA modification signature was correlated with the RNA modification enzymes, so we performed high-throughput RNA-Seq among mouse tissues to qualify the expression levels of genes involved in RNA modification metabolism. According to the RNA modification detection platform and Modomics [25], 59 genes were chosen to analyze the expression pattern among different tissues (Fig. 2A). The heatmap showed that the expression levels of these RNA modification modifiers varied in different tissues but correlated with the tissue-specific RNA modification signatures (Fig. 2A–G). Specifically, tRNA methyltransferase 10 homologue a (Trmt10a), an S-adenosylemethionine-dependent methyltransferase that installs N1-methylguanosine (m^1^G) in tRNAs at the ninth position [38], showed the highest expression level in mouse testis and the lowest level in the heart (Fig. 2B). Consistent with the Trmt10a expression levels, the m^1^G on tRNA-enriched fragments was coordinately expressed across the six mouse tissues with the highest expression in testis and lowest expression in the heart (Fig. 2C) and a positive linear expression correlation between Trmt10a and m^1^G was identified among all tissues in tRNA-enriched fragments, with *r* = 0.5439 and *p* = 0.00196 (Fig. 2D). In addition, Nsun2, a member of the NOL1/NOP2/SUN domain (NSUN) family that adds m^5^C to RNA cytosines in mammalian RNAs [39, 40], also showed a similar expression pattern with Trmt10a in mouse tissues (Fig. 2E), and a linear correlation between Nsun2 and m^5^C for tRNA-enriched fragments was also identified across different tissues (Fig. 2F, G). Interestingly, when comparing the levels of m^1^G and m^5^C on tRNA-enriched fragments across all tissues, we found that the m^1^G and m^5^C have a strong positive linear correlation with *r* = 0.9133 and *p <* 0.0001 (Fig. 2H). This linear correlation was extensively identified in total RNA (*r* = 0.9732 and *p <* 0.0001), mRNA (*r* = 0.9484 and *p* < 0.0001), and 17–50-nt sncRNAs (*r* = 0.8906 and *p* < 0.0001) (Fig. 2I–K), indicating that the expression correlation of different RNA modifications might be present in mammalian RNAs.

### Multiple linear-dependent correlations were identified between different RNA modifications in mouse tissues

**Fig. 3:**
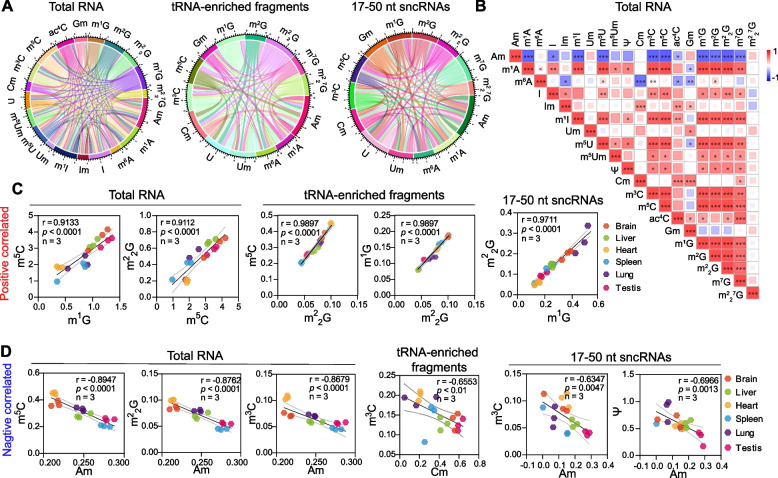
Multiple linear-dependent correlations among different RNA modifications. **A** Circles represent the RNA modifications in total RNA, tRNA-enriched fragments, and 17–50-nt sncRNAs across different mouse tissues (*n* = 3, Additional file 10). **B** Linear correlation analysis between RNA modifications in total RNAs across multiple tissues (brain, liver, heart, spleen, lung, and testis). **C**, **D** The linear correlations of some specific RNA modifications in total RNA, tRNA-enriched fragments, and 17–50-nt sncRNAs. The linear equations and *p* values were shown in each figure. *p < 0.05, **p < 0.01, ***p < 0.001, ****p < 0.0001. The correlation analysis was computed in Pearson correlation coefficients

Coordinate regulation among different epigenetic markers has been reported recently, such as the relationship between DNA methylation and histone marks [41], as well as that between RNA modification and histone marks [42, 43]. However, the regulatory behavior and relationships between different RNA modifications have rarely been investigated. Previous studies have identified a few correlations between RNA modifications, such as the tRNA C38 m^5^C modification, in which the correct addition was dependent on the pre-addition of tRNA 34 queuosine (Q) [44]. By using a high-fat diet-induced metabolic disorder mouse model, we also identified that the level of tsRNA m^5^C was correlated with the level of N2-Methylguanosine (m^2^G) on tsRNA, and deletion of the m^5^C methyltransferase Dnmt2 decreased the level of m^5^C on tsRNA and coordinately downregulated the level of m^2^G on tsRNA [45]. Recently, Ontiveros et al. found that the tRNA m^1^G methyltransferase Trmt10a could interact with mRNA m^6^A demethylase FTO and influence the methylation level of m^6^A on a subset of mRNAs [46]. Therefore, we systematically analyzed the co-expression relationship among different types of RNA modifications across mouse tissue in total RNAs, tRNA-enriched fragments, and 17–50-nt sncRNAs. The circles and correlation matrix showed that many RNA modifications in mouse tissues exhibited strong linear-dependent correlations (Fig. 3 and Additional file 2: Fig. S2). Overall, most of the RNA modifications exhibited a positive linear correlation with other types of modifications (Fig. 3B, C and Additional file 2: Fig. S2A-B), such as m^5^C, m^1^G, m^2^G, N^2^, N^2^-dimethylguanosine (m^2^_2_G), while a small portion of RNA modifications showed a negative linear correlation (Fig. 3B, D and Additional file 2: Fig. S2A-B), such as 2′-O-methyladenosine (Am) in total RNAs and 17–50-nt sncRNAs, and N3-methylcytidine (m^3^C) in tRNA-enriched fragments. Among them, the correlation between m^1^G, m^2^G, and m^2^_2_G, together with m^5^C, showed the strongest positive correlation across all RNA groups in mouse tissues (Fig. 3B–D and Additional file 2: Fig. S2B-D). In addition, m^1^A also showed a significant positive correlation with m^5^C, m^3^C, and RNA methylation on guanosine in total RNAs, tRNA-enriched fragments, and 17–50-nt sncRNAs (Fig. 3B and Additional file 2: Fig. S2A-B). Interestingly, compared to m^1^A, m^6^A did not show a strong correlation with other modifications in total RNA and tRNA-enriched fragments; however, relatively strong correlations of m^6^A with m^5^C, m^1^G, m^2^G, and m^2^_2_G were found in 17–50-nt sncRNAs (Fig. 3B and Additional file 2: Fig. S2A-B). Consistent with previous studies [46], we found that m^6^A in mRNA indeed exhibited a strong correlation with the m^1^G level in tRNA-enriched fragments, and the levels of m^2^G and m^5^C in 17–50-nt sncRNAs also showed a strong positive linear correlation with *r* = 0.9856 and *p* < 0.0001 (Additional file 2: Fig. S2C). There was no tissue specificity of these correlations between different types of RNA modifications across all groups of RNAs (Fig. 3C, D and Additional file 2: Fig. S2C-D), indicating that the coordinated regulation of RNA modification levels in mammals might be prevalent and that the underlying mechanism needs further investigation.

### RNA modification profiles in mouse tissues are sensitive to environmental hypobaric hypoxia exposure

**Fig. 4:**
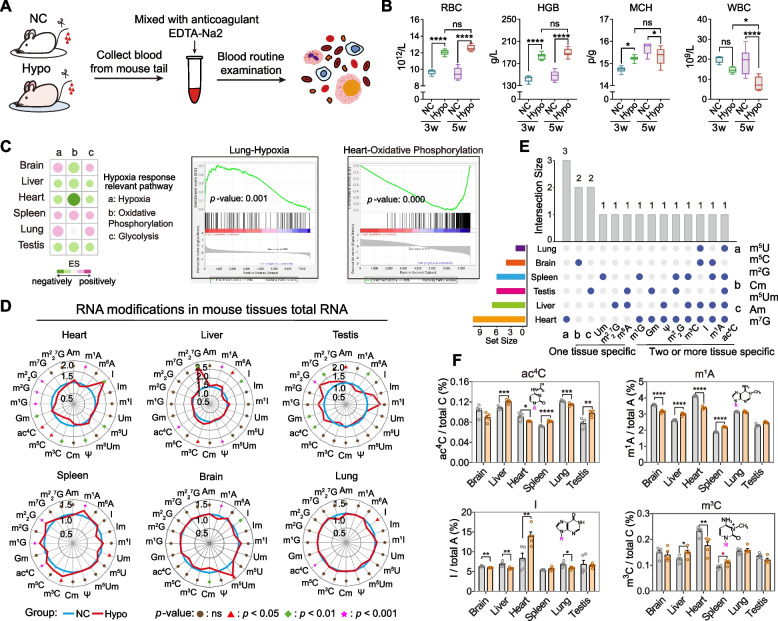
Altered signature of RNA modifications in mouse tissue total RNA by hypobaric hypoxia exposure. **A** Schematic diagram of blood routine analysis. **B** Levels of RBC (red blood cell), HGB (hemoglobin), MCH (mean corpuscular hemoglobin), WBC (white blood cell in peripheral blood) (*n* = 5~10, Additional file 12). **C** The heatmap shows the hypoxia response relevant pathway of different tissues. **D** The retar graph shows the relative RNA modification levels in total RNA between normal (NC) and hypoxia (Hypo) groups across different tissues (*n* = 3~5, Additional files 10 and 11). The level of each RNA modification in the NC group was considered as 1. **E** Distribution of RNA modification types shows a significant difference in tissue total RNA between NC and Hypo groups (*n* = 3~5, Additional files 10 and 11). **F** Comparison of RNA modifications in different tissues’ total RNA between NC and Hypo groups (*n* = 3~5, Additional files 10 and 11). *p < 0.05, **p < 0.01, ***p < 0.001, ****p < 0.0001, ns. not significant. All results are shown as mean ± SEM. Statistical analysis was conducted using Student’s *t*-test with unpaired

Multiple tissues and organs are pathologically activated to adapt to the harsh high-altitude hypobaric hypoxic environment [6, 47]. In fact, the epitranscriptome, including RNA modifications, was sensitive to the environmental exposure (oxidative stress, heat shock, UV-light and chemical stimulation [48], and hypoxia exposure for cultured cells; extremely cold climate and plateau hypoxia for organisms) [35, 49–52] and actively involved in environment adaptation. To further explore the alteration signature of RNA modifications in mouse tissues under hypobaric hypoxia exposure, we simulated a high-altitude hypoxic environment by using a hypobaric chamber with a chamber pressure corresponding to an altitude of 5500 m (Fig. 4A). In the hypoxia group, the number of mouse peripheral red blood cells (RBC) was significantly increased both at 3 weeks and 5 weeks, along with an increase in the hemoglobin concentration (HGB) and mean corpuscular hemoglobin (MCH) (Fig. 4B), which was consisted with a previous study [53]. We also observed the downregulation of the peripheral white blood cell (WBC) numbers, while the red blood cell volume was not significantly affected by the hypoxia exposure (Additional file 3: Fig. S3A). Moreover, the transcriptome sequencing of mouse tissues (brain, liver, heart, spleen, lung, and testis) from the normoxia group (NC) and hypoxia group (Hypo) revealed the activation of hypoxia signaling pathway in the brain, spleen, and lung and the dysregulation of oxidative phosphorylation and the glycolysis pathway across six mouse tissues (Fig. 4C and Additional file 3: Fig. S3B), suggesting that global hypoxia adaptation was activated in response to the high-altitude hypobaric hypoxic environment [54]. Responding to the activation of hypoxia adaptation, the RNA modification signature of mouse tissues on total RNA was significantly altered and varied across different tissues (Fig. 4D–F and Additional file 3: Fig. S3C). The heart (with 11 types of altered RNA modifications) and liver (with 7 types of altered RNA modifications) showed the most dynamic regulation on total RNA modification levels (Fig. 4D, E). Among all the RNA modifications, the m^2^_2_^7^G and N7-methyleguanosine (m^7^G) were uniquely upregulated in the liver and testis, respectively, while the 2′-O-methyluridine (Um) was specifically downregulated in the spleen and the 5-methyluridine (m^5^U), m^5^C, m^2^G were selectively downregulated in the heart (Fig. 4D, E), indicating that a diverse regulatory mechanism in hypoxia adaptation may occur in different tissues. In addition, few RNA modification types, such as the N4-acetylcytidine (ac^4^C), m^1^A, I, and m^3^C, showed extensive alterations across multiple tissues (Fig. 4F), which might suggest a general molecular reaction of the organism under hypoxia adaptation. Interestingly, compared to the significant alterations in RNA modification levels under a hypoxic environment, the overall alterations at the transcriptome level were moderate under hypoxia exposure in most tissues except for the heart (Additional file 4: Fig. S4). Taken together, our data demonstrated that hypoxia stress could induce dynamic alteration of the RNA modification signature in mouse tissues. These alterations are tissue specific, indicating that RNA modifications might play different potential roles in high-altitude adaptation regulation across tissues.

### Hypobaric hypoxia induced alteration to overall tRNA modification in the mouse testis

**Fig. 5:**
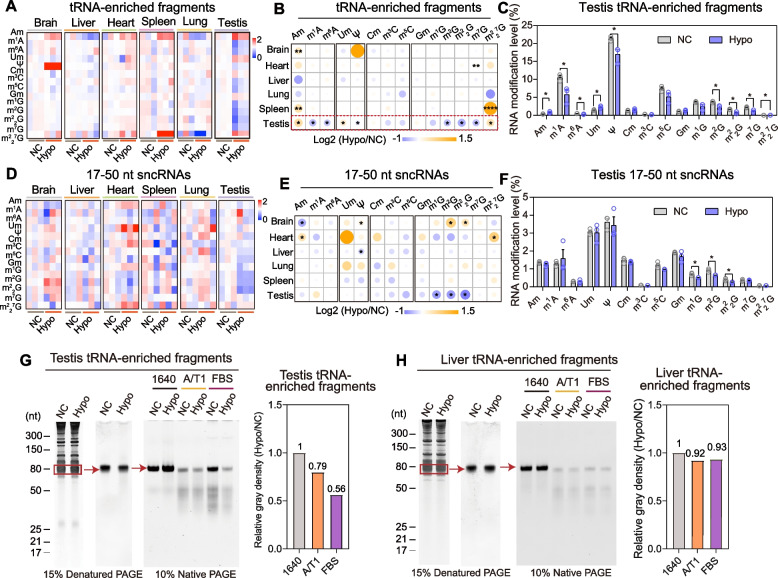
Altered signature of RNA modifications in mouse tissue tRNA-enriched fragments and 17–50-nt sncRNAs by hypobaric hypoxia exposure. **A**, **D** Fold changes of RNA modification levels on tRNA-enriched fragments (**A**) and 17–50-nt sncRNAs (**D**) from NC and Hypo group mouse tissues (*n* = 3, Additional files 10 and 11). The value > 0 indicates that the levels of modification were significantly increased under hypoxia environment, while the value < 0 means that the levels of RNA modification were significantly decreased under hypoxia exposure. **B**, **E** The comparison of RNA modification levels in tRNA-enriched fragments (**B**) and 17–50-nt sncRNA (**E**) across multiple tissues between NC and Hypo groups. **C**, **F** RNA modification level on testis tRNA-enriched fragments (**C**) and 17–50-nt sncRNA (**F**) (*n* = 3, Additional files 10 and 11). **G**, **H** Isolation of tRNA-enriched RNA fragments from testis (**G**) and liver (**H**) and their stability against RNase A/T1 and fetal bovine serum (FBS). The gray analysis showed the average value of three repeated experimental data that calculated by using Fluor ChemHD2. The level of tRNA in the 1640 group was considered as 1. *p < 0.05, **p < 0.01, ****p < 0.0001. Statistical analyses were conducted using Student’s *t*-test unpaired

Total RNA in mouse tissue consists of a large amount of rRNA (over 80%), with some tRNA (~10–15%), noncoding RNA, and mRNA (~2–5%) [55]. tRNA, as the most extensively modified RNA, is the key regulator of mRNA decoding and cellular translational control [23]. Since tRNA modifications play a key role in maintaining the higher structure of tRNA and its biological functions [24], we next focused on identifying the alteration signature of RNA modification levels on tRNA-enriched fragments and 17–50-nt sncRNA in mouse tissues to explore the RNA modification-dependent molecular adaptation under hypobaric hypoxia exposure. Surprisingly, out of line with our expectation, the overall total tRNA modifications in most detected tissues were slightly altered under a hypobaric hypoxic environment (Fig. 5A, B), and Am and m^2^_2_^7^G showed the most dynamic regulation across different tissues (Fig. 5B). However, in tRNA-enriched fragments from the testis, we found that the levels of 9 types of RNA modifications were significantly affected by the hypoxic environment (Fig. 5B, C). In addition, similar to tRNA-enriched fragments, the RNA modification patterns of the 17–50-nt sncRNAs in different tissue were mostly unaffected by hypoxia exposure (Fig. 5D, E). Only 4 and 3 types of RNA modification levels in 17–50-nt sncRNAs were significantly changed in the brain and testis under hypoxia exposure, respectively (Fig. 5E, F).

### Reprogramming of the tRNA modification pattern under hypobaric hypoxia affects tRNA stability against RNase

To investigate the effect of hypobaric hypoxic environment-induced RNA modification alteration on tRNA stability, we isolated the total tRNA from the testes of mice under either hypobaric hypoxia exposure or normoxic environment to analyze tRNA stability against RNase A/T1 (a mixture of RNase A and RNase T1, in which RNase A specifically cleaves RNA at C and U residues and RNase T1 specifically hydrolyzes RNA at G residues) and fetal bovine serum (FBS, which contains a mixture of various RNase) [45]. The results showed that the tRNA-enriched fragments from the testis in the hypoxia group were rapidly degraded after RNase A/T1 and FBS treatment, showing much more RNase sensitivity than those in the normoxia group (Fig. 5G). However, the RNase sensitivity of tRNA-enriched fragments from the liver was similar between the normoxia group and the hypoxia group (Fig. 5H), which may be related to the slightly altered RNA modification level in mouse liver total tRNA under hypobaric hypoxia exposure (Fig. 5A, B).

**Fig. 6:**
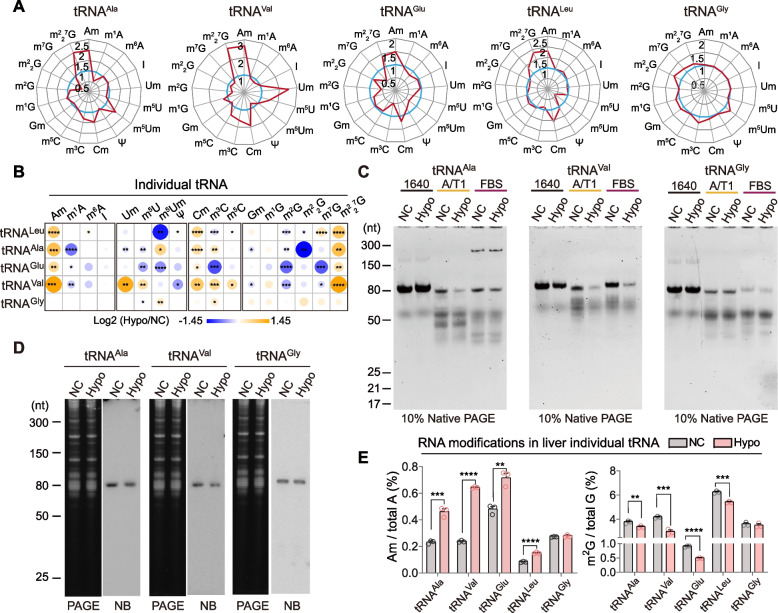
Altered signature of RNA modifications in liver individual endo-tRNA by hypobaric hypoxia exposure. **A** The significant difference of RNA modifications in liver individual endo-tRNA^Ala^, tRNA^Val^, tRNA^Glu^, tRNA^Gly^, and tRNA^Leu^ between NC and Hypo groups. **B** The retar graph shows the altered pattern of RNA modifications in liver individual endo-tRNA^Ala^, tRNA^Val^, tRNA^Glu^, tRNA^Gly^, and tRNA^Leu^ between NC and Hypo groups (*n* = 3, Additional files 10 and 11). **C** Stability of liver individual endo-tRNA^Ala^, tRNA^Val^, and tRNA^Gly^ against RNase A/T1 and fetal bovine serum (FBS). **D** Expression levels of individual endo-tRNA^Ala^, tRNA^Val^, and tRNA^Gly^ in the liver, which showed the represent northern blot result of three times independent repeats. **E** The levels of Am and m^2^G in liver individual endo-tRNA^Ala^, tRNA^Val^, tRNA^Glu^, tRNA^Leu^, and tRNA^Gly^ (*n* = 3, Additional files 10 and 11). *p < 0.05, **p < 0.01, ***p < 0.001, ****p < 0.0001; NB, northern blot. All results are shown as mean ± SEM. Statistical analysis was conducted using Student’s *t*-test unpaired

To further investigate the effect of hypobaric hypoxia exposure on individual tRNAs, we randomly selected several individual endo-tRNAs with high codon usage (Additional file 5: Fig. S5A) to analyze the alterations of the individual tRNA modification landscape under hypobaric hypoxia exposure. Since the isolation of a single tRNA from total RNA requires a large input of total RNA, we only chose the liver for this experiment in the present study; five individual tRNAs (tRNA^Ala^, tRNA^Val^, tRNA^Glu^, tRNA^Gly^, and tRNA^Leu^) were successfully isolated from mouse liver total RNA in the hypoxia group and normoxia group (Additional file 5: Fig. S5B-C). Notably, diverse alteration patterns of tRNA modifications in the hypoxia group were observed in these isolated liver endo-tRNAs (Fig. 6A, B). More than half of the detected types of RNA modifications were significantly dysregulated in tRNA^Ala^, tRNA^Val^, tRNA^Glu^, and tRNA^Leu^ under hypoxia exposure, while only 3 types of RNA modifications were identified as significantly altered on tRNA^Gly^ (Fig. 6B). Consisted with the alteration of RNA modification levels under hypoxia exposure, the tRNA^Ala^ and tRNA^Val^ exhibited much more sensitivity to RNase A/T1 and FBS degradation in the hypoxia group, while tRNA^Gly^ showed similar sensitivity to RNase treatment in the normoxia group and the hypoxia group (Fig. 6C). Accordingly, the expression levels of tRNA^Ala^ and tRNA^Val^ significantly decreased in the mouse liver in the hypoxia group, while the level of tRNA^Gly^ showed no change between the normoxia group and the hypoxia group (Fig. 6D), consistent with the tRNA modification alteration pattern and RNase sensitivity under hypoxia exposure (Fig. 6A, B and E).

Interestingly, we found that some types of RNA modifications were simultaneously regulated in different individual tRNAs in the same tissue (Fig. 6A, E). For example, Am, Cm, and m^2^_2_^7^G were significantly upregulated in liver tRNA^Ala^, tRNA^Val^, tRNA^Glu^, and tRNA^Leu^ in the hypoxia group, while the m^2^G was downregulated (Fig. 6E), which might be due to the tissue-specific regulation of RNA modification modifiers in hypoxia adaptation. In summary, our data demonstrated that the dysregulation of the RNA modification landscape on mouse tissue tRNA in a hypobaric hypoxic environment affected the tRNA epitranscriptome at both tRNA modification level and tRNA repertoire. These results further suggest an active role of tRNA modifications in high-altitude hypoxia adaptation.

### Hypobaric hypoxia-induced tRNA epitranscriptome alteration attenuates cellular translation and cell proliferation

**Fig. 7:**
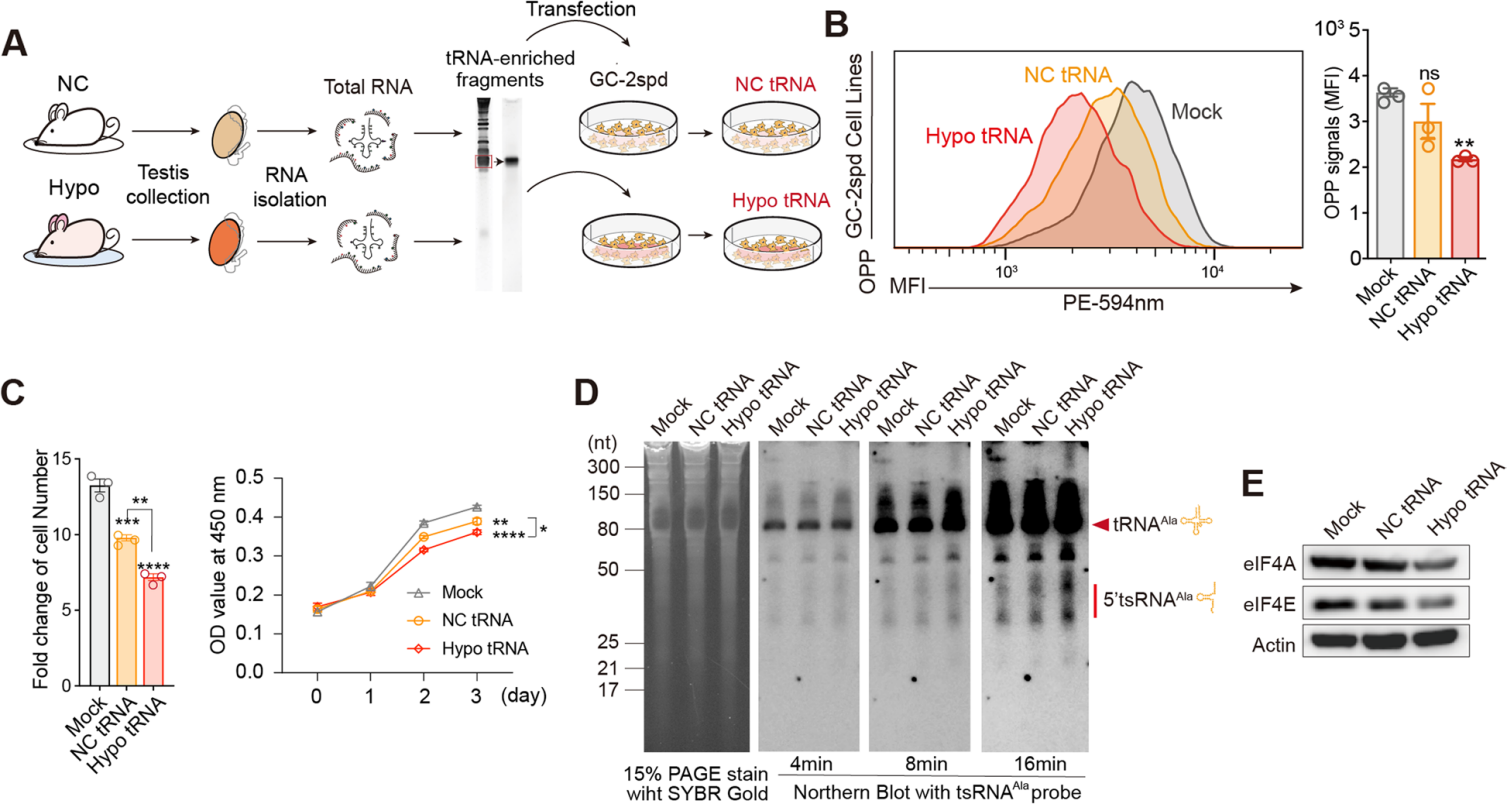
Functional analysis of total tRNA from NC and hypoxia groups’ testis in GC-2spd cells. **A** Schematic diagram of testis total tRNA-enriched fragment transfection into GC-2spd cells. **B** Nascent protein synthesis of GC-2spd cells transfected with total tRNA-enriched fragments from NC and Hypo group mouse testis. **C** Growth analysis of GC-2spd cells through counting cell number at 48 h after transfection and CCK8 at pointed time after transfection (*n* = 3~7, Additional file 13). **D** Expression levels of individual tRNA^Ala^ and tsRNA^Ala^ in GC-2spd cells transfected with total tRNA-enriched fragments from NC and Hypo group mouse testis after 48 h. **E** Western blot analysis of eIF4A and eIF4E expression in GC-2spd cells at 48 h transfected with total tRNA between NC and Hypo groups from mouse testis. **p < 0.01, ***p < 0.001, ****p < 0.0001; NB, northern blot. All results are shown as mean ± SEM. Statistical analysis was conducted using one-way ANOVA with uncorrected Fisher’s LSD

Since tRNAs are the fundamental component of cellular translational machinery and have a central role in translation [23], the dysregulation of the tRNA epitranscriptome under hypobaric hypoxia exposure might affect the cellular translation rate in response to hypoxia adaptation. To confirm this, we isolated total tRNA from the testis of mice either under normoxic conditions or hypoxia exposure and transfected these tRNA-enriched fragments into GC-2spd cells, a mouse spermatocyte cell line [56] (Fig. 7A). No significant transcriptomic-level alterations were observed between the normoxic tRNA transfection group and the hypoxic tRNA transfection group (Additional file 6: Fig. S6), which was consistent with the transcriptome alterations in mouse testes under hypoxia exposure (Additional file 4: Fig. S4F). As expected, the transfection of hypoxia group tRNA-enriched fragments from the testis indeed led to a significant reduction in overall nascent protein synthesis in GC-2spd cells (Fig. 7B), demonstrating that the dysregulation of tRNA epitranscriptome by hypoxia exposure might play a role in reducing the translational rate in cells. Moreover, the cell proliferation rate of GC-2spd cells was also affected by the transfection of tRNA-enriched fragments from the testis, in which significant inhibition of the cell proliferation rate was observed in NC group, and a more severe cell proliferation inhibition was observed in the Hypo tRNA transfection group (Fig. 7C).

tsRNAs have versatile regulation mechanisms in various fundamental biological processes. Some evidence has suggested the function of tsRNAs lies in cellular translational control by competing with their mature tRNA precursors for ribosomal loading or by competing with mRNAs for translation initiation factor binding to control the overall translation rate in cells [32, 57]. 5′ tsRNA^Ala^ has been reported to displace the translation initiation factor eIF4E/G/A or PABPC1 from m^7^G-capped mRNAs and induce the reduction in overall translation in cells [58]. In tRNA-enriched fragment-transfected cells, we observed an increase in 5′ tsRNA^Ala^ (Fig. 7D) and the downregulation of translation initiation factors eIF4A/E in hypo tRNA-transfected group (Fig. 7E), which might work cooperatively with extra tsRNAs to reduce the translation in tRNA-enriched fragment-transfected cells. Although there was no direct evidence showing how the altered tRNA epitranscriptome under hyperbaric hypoxia exposure affects cellular translation rate in mouse tissue, our data provides new insights and opens opportunities to investigate how RNA modification and the tRNA epitranscriptome reprogram translation in response to high-altitude hypoxia adaptation.

## Discussion

As a novel epigenetic marker, RNA modification landscape in mouse tissue under exposure to diverse environment has been less characterized. Accumulating evidence from recent decades has revealed how RNA modifications affect RNA metabolism and protein homeostasis in cells [14, 59, 60]. This study, for the first time, systematically characterized the RNA modification landscape of different RNA types across mouse brain, liver, heart, spleen, lung, and testis and revealed the diversity of RNA modification signature in different tissues, suggesting a tissue-specific epitranscriptome beyond DNA sequence. Total RNA from mouse tissue consists of different classes of RNAs, such as rRNAs, mRNAs, tRNAs, and noncoding RNAs. Theoretically, the RNA modification levels revealed in total RNA represent the overall RNA modification signature of all RNAs. However, because over 80% of the total RNA is rRNA [37] and rRNA is enriched in RNA modifications [21], the RNA modification pattern of total RNA in mouse tissue might largely represent the RNA modification signature of rRNAs. Moreover, since single-stranded RNA marker-based gel excision was used to classify different RNA fragments, the isolation of specific RNA fragments in this study is actually a mixture of RNAs with similar nucleotide length ranges. The quantified abundance of RNA modifications is a reflection of different RNAs contained in each RNA group, which impacts our understanding of the RNA modification profile of a specific class of RNAs. For example, the 17–50-nt sncRNAs are composed of miRNAs, tsRNAs, rsRNAs, piRNAs, ysRNAs, and other types of sncRNAs, and the RNA modification signature is an overall reflection of these RNAs. Moreover, since the snoRNA shared a similar nucleotide length with mature tRNA, the tRNA-enriched fragments might be contaminated with a small portion of snoRNAs, which might also contribute a little to the alteration of RNA modification of tRNA-enriched fragments under hypoxia exposure and the cellular outcomes of tRNA-enriched fragment transfection into GC-2spd cells.

The RNA modifications range from simple methylation to complex multistep chemical modifications in tRNA, such as m^5^C, m^1^A, and N6-threonylcarbamoyladenosine (t^6^A) [61]. To date, more than 170 types of RNA modifications have been identified across three kingdoms in nature [25]. Due to the availability of modified RNA nucleobase standard and the frequency of different types of RNA modifications, we only applied 22 types of modified nucleobase standards in our RNA modification detection platform to qualify the expression levels of these RNA modifications in mouse tissue RNA, which limited our ability to obtain a more extensive landscape of RNA modification signatures under hypobaric hypoxia exposure. The 3-methyluridine (m^3^U) and 5-hydroxymethylcytidine (hm^5^C) were not precisely quantified in all types of RNAs. Although a previous study reported that Tet2 could modify tRNA with hm^5^C and regulate the biogenesis of tsRNAs [62] and that bmt5/6 is responsible for the m^3^U methylation of 25S rRNA in yeast [63], the low signal-to-noise ratio (SNR) of hm^5^C and m^3^U in mouse tissue RNAs made it difficult to quantify across all RNA types. Interestingly, we found that m^5^C, m^1^A, and Ψ compose over 50% of the quantified RNA modifications in tRNA. m^5^C and Ψ are present at multiple nucleotide sites within a signal tRNA, while most RNA modifications exist at one or two specific positions, which may explain the high levels of m^5^C and Ψ in tRNA [64].

Hypoxia is one of the most important factors affecting body health when entering high-altitude areas. The high-altitude-induced dysregulation of the hematopoietic system, especially excessive erythrocytosis, has been well investigated previously; these mechanisms are associated with genetic adaptation [7], reactivity to hypoxia-induced erythropoietin (EPO), the delivery of iron, the regulation of hypoxia-inducible factor-1 (HIF-1) [65], and the prolonged half-life of peripheral red blood cells [66]. A study also reported the EPO-independent hematopoietic stem cell (HSC) mechanism of high-altitude erythrocytosis [53]. By using a similar hypobaric hypoxia mouse model, they found that hypoxia exposure induced an expansion of HSCs in vivo and disturbed the lineage choice decisions of HSCs, which further led to the dysregulation of the hematopoietic system and impacted the composition of peripheral blood cells [53]. As the key regulator of the epitranscriptome, RNA modifications, such as m^6^A [67], m^5^C [68], and A-to-I [69], were reported to be involved in regulating hematogenesis under physiological conditions. However, few studies have examined the dysregulation of hematogenesis under high-altitude environment exposure due to RNA modifications. The dysregulation of the abundance of RNA modifications in multiple tissues under hypoxia exposure might provide new insight into the underlying mechanism of high-altitude adaptation in hematogenesis.

The abundance of RNA modifications is determined by the target RNA levels of various RNA modification modifiers [17]. The observed correlation between RNA modification abundance and RNA modifier levels in a tissue-specific manner might suggest a different expression pattern of RNA modification regulators across tissues for the establishment of tissue-specific biological functions. Moreover, our data also showed that the levels of multiple RNA modifications exhibited a strong linear correlation with each other across mouse tissues, indicating that a coordinated regulatory network of RNA modification regulators was established to maintain the homeostasis of the tissue-specific epitranscriptome and physiological function. Identification of the tissue-specific RNA modification signature and the correlation between diverse RNA modifications might open new opportunities for investigation into the multifaceted regulating function and mechanism of epitranscriptome in defining tissue specificity.

Since emerging evidence has shown that many RNA modification sites in tRNA are not fully modified and can be dynamically regulated in a cell type- and cell state-dependent pattern [30], the concept of the tRNA epitranscriptome or tRNA code was raised to demonstrate tRNA heterogeneity in cells [24]. Our data suggested that this concept was valid by identifying the tissue-specific tRNA modification signature and its dynamic regulation in mouse tissue under hypobaric hypoxia exposure. Indeed, by using the present methods, it is difficult to pinpoint the site-specific RNA modification in mouse tissue and characterize the alteration of the RNA modification signature under environmental exposure at single-nucleotide resolution. However, the incoming technology MLC-Seq has been developed to allow for the simultaneous identification of the tRNA sequence and mapping of the tRNA modification at specific sites [70]. This application will give researchers a more comprehensive overview of the alteration landscape of the tRNA epitranscriptome, as well as the regulatory mechanism of how tRNA epitranscriptome reprograms cellular translation in high-altitude hypoxia adaptation.

Notably, the fascinating roles of tRNA modifications on tRNA function and tsRNA biogenesis under hypobaric hypoxia exposure are still not clear. Transfection of tRNA-enriched fragments from mouse testis in the hypoxia group into GC-2spd cells led to a decreased protein synthesis rate and cell proliferation rate with an increased tRNA fragmentation rate, suggesting a deleterious role of hypoxic tRNA in spermatocytes. However, since the GC-2spd cell line is an engineered spermatocyte cell line with a rapid cell proliferation rate, it should be noted that the results from GC-2spd cells cannot mimic spermatocytes under normal physiological conditions. Moreover, the transfection of tRNA-enriched fragments from the normoxia group also induced a similar effect as those from the hypoxia group in GC-2spd cells. The reason could be explained as follows: the input of extra tRNA might disturb the balance of the in vivo tRNA pool in GC-2spd cells, which further activates the tRNA degradation pathways to remove the partially processed, hypomodified or misfolded tRNAs in cells. The disturbed tRNA degradation pathway then alters the tRNA pool in cells and affects the cell translation rate, finally attenuating the cell proliferation rate. In addition, as a result of tRNA fragmentation, more tsRNAs are produced in cells, which might also contribute to the slowdown of the translation rate in GC-2spd cells via multiple pathways [32]. Collectively, our study suggested that hypobaric hypoxia-induced alterations in the tRNA modification landscape destabilized tRNA and increased the level of tsRNA, which further led to the reduction in the cellular translation rate, suggesting that tRNA modifications might be involved in hypoxia adaptation in a tsRNA-dependent manner. However, which tsRNAs were responsible for the overall translation rate reduction and which proteins were affected in response to hypoxia adaptation are still unclear. Answers to these questions will expand our knowledge of the roles of the tRNA epitranscriptome and tsRNAs in environment-dependent cell-specific translational control and the adaptation mechanisms on high-altitude hypobaric hypoxia.

## Conclusions

In summary, our study revealed the tissue-specific RNA modification signature across mouse tissues and provided comprehensive analyses of the alterations to RNA modifications abundance under hypobaric hypoxia exposure, which highlights the molecular complexity of RNA modifications to facilitate high-altitude adaptation of tissues.

## Methods

### Animal experiments

Eight-week-old male C57BL/6J mice purchased from Tengxin Biotechnology Co., Ltd (Chongqing) were randomly divided into the normoxia control group and the hypoxia group. Mice in the hypoxia group were exposed to a hypoxic environment for 5 weeks by being raised in the hypobaric chamber with chamber pressure that corresponded to an altitude of 5500 m. Mice in the normoxia group were raised at a 300-m altitude in IVC cages for 5 weeks. All the mice were raised in a controlled ambient temperature of 23 ± 3 °C and 40–70% relative humidity with a 12-h light/12 h dark cycle and allowed free access to food and water. Three weeks and 5 weeks after hypoxia or normoxia exposure, peripheral blood was collected from the tail and placed in a centrifuge tube containing EDTA anticoagulant, and routine blood analysis was determined by using an animal automatic blood cell analyzer (MindrayBc-5300). All animal experimental manipulations were in accordance with the Guidelines for Animal Care of China and approved by the Ethics Committee of Army Medical University.

### Sample collection and total RNA extraction

Following 5 weeks of hypoxic challenge, the brain, lung, heart, liver, testis, and spleen were collected and immediately frozen in liquid nitrogen for RNA extraction. Total RNA was extracted with RNAiso Plus reagent (Takara, catalog number: 9108) according to the manufacturer’s protocol. Briefly, 1 ml RNAiso Plus was added to a microtube with pulverized tissues or cells and vortexed vigorously, and the sample was incubated for 5 min at room temperature. Then, 200 μl chloroform was added to the above sample, and it was vortexed and incubated for 10 min at room temperature, followed by centrifuging at 12,000 × g for 15 min at 4 °C. The supernatant was collected into a new microtube and mixed with an equal volume of isopropanol. After gently mixing, the mixture was incubated for 10 min at room temperature and centrifuged at 12,000 × g for 15 min at 4 °C. Finally, the RNA pellet was washed with 75% ethanol and resuspended with RNase-free water.

### Isolation of Poly (A)-enriched RNAs

Poly (A)-enriched RNAs were isolated from total RNA using Poly(A)mRNA Magnetic Isolation Kit (NEB, catalog number: E7490L) according to the manufacturer’s protocol. In brief, 5 μg of DNA-free total RNA was diluted with RNase-free water to a final volume of 50 μl in a 0.2-ml PCR tube. Transfer 30 μl of well-resuspended NEB Next Magnetic Oligo d(T) beads into a new PCR tube and wash the beads twice with RNA binding buffer. Next, add 50 μl RNA binding buffer to the 50 μl total RNA and mix thoroughly. The mixture was heated at 65 °C for 5 min to denature the RNA and facilitate the binding of the poly-A-RNA to the beads. Resuspend the beads using the RNA-RNA binding buffer and incubate for 10 min at room temperature, and repeat once again after mixing. Then, place the tubes on the magnetic rack at room temperature for 2 min and remove all of the supernatants. Wash the beads twice by adding 200 μl of wash buffer to remove unbound RNA. Add 50 μl Tris Buffer to each tube and gently pipette to mix thoroughly. The samples were heated at 80 °C for 2 min to elute the poly(A) RNA from beads. A total of 50 μl binding buffer was directly added to the above tube and the mixture was incubated at room temperature for 10 min. Place the tubes on the magnetic rack at room temperature for 2 min and discard all of the supernatants. Wash the beads once with 200 μl of wash buffer and elute Poly (A)-enriched RNAs using 50 μl Tris Buffer twice. Collect the purified Poly (A)-enriched RNAs by transferring the supernatant to a clean nuclease-free PCR tube and precipitate RNA.

### Isolation of ~80-nt and 17–50-nt RNA fragments from total RNAs

A total of ~80-nt and 17–50-nt RNA fragments were separated and isolated on 15% denatured PAGE gel according to the RNA marker. Briefly, 3 μg total RNA was electrophoresis on 15% Urea-PAGE for 1 h in 1XTBE buffer (Invitrogen, catalog number: B540024-0001) at 200 V. After staining the gel by SYBR GOLD (Invitrogen, catalog number: S11494), ~80-nt and 17*–*50-nt RNA fragments were excised and recycled from the gel according to RNA Ladder (NEB, catalog number: N0364S and N2102S) as we previously reported [45].

### Tissue transcriptome sequencing and data analysis

After RNA extraction, transcriptome sequencing was commissioned by Shanghai Personalbio Technology Co., Ltd (Shanghai, China) and BGI (ShenZhen, China). Briefly, mRNAs with polyA tails were enriched by Oligo (DT) magnetic beads from total RNA. Then, mRNA was randomly interrupted, linked with connectors, and reverse transcribed to cDNA for library construction. Finally, pair-end sequencing was conducted based on a high-throughput sequencing platform. Furthermore, the raw sequencing data were filtrated by Cutadapt [71], spliced high-quality sequence fragments using Hisat2 [72], and finally quantified by StringTie [73]. The differentially expressed genes were screened by using R package edgeR [74]. GO enrichment analysis and GSEA enrichment analysis were performed using R package clusterProfiler [75] and GSEA software [76], respectively. Gene sets were considered statistically enriched if the normalized *p* value < 0.05.

### Detection and quantitative analysis of RNA modifications by LC-MS/MS

The RNAs were digested into mononucleotides by using a previously reported procedure [45]. Briefly, 100 ng RNAs were digested in a 30-μl reaction system containing 3 μl 10 × RNA hydrolysis buffer (Tris-HCl 2500 mM, pH 8.0; 50 mM MgCl2 and 5 mg/mL BSA), 1 IU benzonase (Sigma-Aldrich, catalog number: E8263), 0.2 IU alkaline phosphatase (Sigma-Aldrich, catalog number: P5521), and 0.05 IU phosphodiesterase I (USB, catalog number: 20240Y) at 37 °C for 3 h. Then, the enzymes in the digestion mixture were removed using a Nanosep 3K spin filter (Pall Corporation, catalog number: 0D003C34). Before transferring the digested RNA samples, the spin filter was rinsed twice with 400 μl distilled water at 14,000 × g, for 15 min at room temperature. At last, processed RNAs were stored at −20°C for LC-MS/MS analysis. The LC-MS/MS-depended RNA modification detection experiments were performed based on our previously reported methods by using waters ACQUITY-UPLC I-class carrying Xevo-TQ-S mass spectrometry system [45]. The expression level of each RNA modification in this study was quantitatively calculated according to the standard curve and our previously reported RNA modification relative quantification methods [45]. The modified nucleotide standards used in this study are listed in Additional file 1: Fig. S1A.

### Isolation of liver individual tRNA

To isolate single tRNA^Ala^, tRNA^Val^, tRNA^Glu^, tRNA^Leu^, and tRNA^Gly^, small RNA (< 200 nt) was extracted using RNAiso for Small RNA Kit (Takara, catalog number: 9753A) according to the manufacturer’s instruction. One milligram of small RNAs was then incubated overnight at 50°C with Biotin-labeled oligonucleotides probes (5′-tRNA^Ala^: 5′-Bio-CGCTCTACCACTGAGCTACACCCCC; 5′-tRNA^Val^: 5′-Bio-GTGATAACCACTACACTACGGAAAC; 5′-tRNA^Glu^: 5′-Bio-ATCCTAACCGCTAGACCATGTGGA; 5′-tRNA^Gly^: 5′-Bio-AATTCTACCACTGAACCACCCATGC; 5′-tRNA^Leu^: 5′-Bio-) CCTTAGACCGCTCGGCCATCCTGAC) in the 1 × SSC buffer. Following overnight incubation, the RNA solution was incubated for 30 min at room temperature with streptomycin affinity agarose beads (Cytiva, catalog number: 17511301) which were washed three times using 400 μl 20 uM Tris-HCl (PH = 7.5) in ULTARAFREE MC GV STER tube (Millipore, catalog number: UFC30GV0S) in advance. The mixture was centrifuged at 2500 g for 30 s and the centrifugal liquid was collected as a negative control, followed by a wash with 0.5 × SSC. Finally, targeted tRNAs were washed from the beads with RNase-free water and extracted using ethyl alcohol. Isolated individual tRNA was confirmed by the northern blot. Original uncropped images of the northern blot and denatured gel are shown in Additional file 7: Fig. S7.

### tRNA stability analysis

To analyze the stability of tRNA purified from hypoxia mouse tissue, tRNAs (~80 nt RNA fragments) were incubated at 37 °C for 15 min in RPMI 1640-base systems, with three groups set as follows: ① 15 ng RNAs in 9 μl RPMI 1640 (HyClone, catalog number: SH30809.01); ② 15 ng RNAs in 9 μl RPMI 1640 with 0.8% FBS (Clark Bioscience, catalog number: FB25015); and ③ 15 ng RNAs in 9 μl RPMI 1640 with 0.0003 μl RNaseA/T1 (2 mg/ml of RNase A and 5000U/ml of RNase T1). After incubation, the samples were immediately incubated on ice and added with RNA Loading Dye (2 ×) (New England Biolabs Inc., catalog number: B0363S). Four-microliter samples were run in 10% native PAGE gel at 4 °C. The gels were stained with SYBR Gold before imaging. Original uncropped images of the northern blot and denatured urea gel are shown in Additional file 8: Fig. S8.

### Northern blots

Isolated RNAs were denatured at 95°C for 3 min and separated using 15% Urea-PAGE. The gels were then stained with SYBR GOLD, imaged and immediately transferred to Roche Nylon Membranes (Roche, catalog number: 11417240001), and UV-cross-linked twice at 0.12J energy. Membranes were pre-hybridized with Roche DIG hybridization buffer (Roche, catalog number: 11796895001) for 60 min at 42°C shaker. DIG- labeled probes (5′-tRNA^Ala^: 5′-DIG-CGCTCTACCACTGAGCTACACCCCC; 5′-tRNA^Val^: 5′-DIG-GTGATAACCACTACACTACGGAAAC; 5′-tRNA^Gly^: 5′-DIG-AATTCTACCACTGAACCACCCATGC) were added to the hybridization solution and incubated overnight at 42°C. Discarding the hybridization solution, the membranes were washed twice at 42°C with low stringent buffer (2 × SSC with 0.1% [wt/vol] SDS) for 15 min each, followed by twice washes with high stringent buffer (0.1 × SSC with 0.1% [wt/vol] SDS) for 10 min each, and finally washed by washing buffer (1 × SSC) for 10 min. Then, the membrane was blocked in a blocking buffer (Roche, catalog number: 11096176001) at room temperature for 3 h. After which, DIG antibody (Roche, Anti-Digoxigenin-AP Fab fragments, 11093274910) was added into the blocking buffer at a ratio of 1:10,000 and incubated again for another 1 h at room temperature. The membranes were washed 4 times in DIG wash buffer (1 × Maleic acid buffer, 0.3% Tween-20) at room temperature for 15 min each, followed by incubation in developing buffer for 10 min at room temperature. Finally, the membranes were coated with CSPD reagent (Roche, catalog number: 11755633001) at 37°C for 15 min in the dark and imaged by using a Bio-Rad system (USA). Original uncropped images of northern blot and denatured urea gel are shown in Additional file 9: Fig. S9.

### Cell culture

Germ cell-2 spermatid (GC-2spd) cells were cultured in DMEM medium (BI, catalog number: 010521ACS) supplemented with 10% fetal bovine serum (FBS) (BI, catalog number: 04-001-1ACS) and 1% antibiotics (100 U/ml penicillin and 100 U/ml streptomycin) in an incubator (Thermo; Thermo Fisher Scientific Inc, Waltham, MA, USA) containing 21% O_2_ and 5% CO_2_ at 37 °C.

### Transfection of testis tRNA into GC-2spd cell

Seeding 0.5 × 10^6^ GC-2spd cells per well into the 6-well plate and transfection was performed the next day when the cell reached 70*–*90% confluent. Four hundred nanograms of tRNA purified from testis was transfected into each well using Lipofectamine^TM^ 3000 Reagent (Invitrogen, catalog number: 31985062) according to the instructions as previously reported [45]. Before transfection, the culture medium was discarded and a 2-ml fresh DMEM medium was added. Forty-eight hours after transfection, cells were harvested and counted using a Neubauer hemocytometer (Qiujing Industrial Co., LTD, Shanghai, China) to analyze cell proliferation.

### Protein synthesis assay

Protein synthesis assay was performed at 48 h after transfection of testis tRNA to evaluate the effect of RNA modification change on tRNA function using Click-iT® Plus OPP Protein Synthesis Assay Kits (Life technology, catalog number: C10456) according to the manufacturer’s instructions. In brief, dilute Click-iT® OPP (component A) 1:1000 in 1 ml DMEM supplemented with 10% FBS and 1% antibiotics to prepare a 20-μm final working solution; remove the medium, add 1 ml per well of DMEM containing 20 μm Click-iT® OPP, and incubate cells at 37°C for 15 min; after incubation, remove the DMEM, wash the cells once with PBS, and then digest cells with pancreatin; wash cells with PBS and resuspend with 100 μl of 3.7% formaldehyde and incubate for 15 min at room temperature, followed by incubation of 0.5% Triton®X-100 for 15 min at room temperature; then, prepare 1 ml Click-iT® Plus OPP reaction cocktail consisted of 880 μl Click-iT® OPP reaction buffer, 20 μl Copper Protectant (component D), 2.5 μl Alexa Fluor® picolyl azide (component B), and 100 μl Click-iT® Reaction Buffer Additive, remove the permeabilization buffer, wash cells twice with PBS, and add 100 μl of above reaction cocktail to incubate 30 min at room temperature; finally, dye with DAPI for 20 min to perform flow analysis using Kaluza (Beckman Coulter).

### Western blot analysis

Western blot analysis was performed to evaluate the expression level of eukaryotic initiation factor 4A/E (eIF4A and eIF4E). In brief, lysates from cells mixed with 5 × SDS gel loading buffer were run in SDS-PAGE gels. Beta Actin Mouse McAb (Proteintech, catalog number: 60008), eIF4A (C32B4) Rabbit mAb (CST, catalog number: 2013S), and eIF4E (CST, catalog number: 9742S) Rabbit mAb were used as primary antibody. Horseradish peroxidase (HRP)-conjugated goat anti-rabbit immunoglobulin G (Proteintech, catalog number:SA00001:15) and HRP-conjugated goat anti-mouse immunoglobulin G (Beyotime, catalog number: A0216) were used as a secondary antibody. The blots were scanned using the Bio-Rad system (USA). Original uncropped images of the western blot are shown in Additional file 9: Fig. S9.

### Data analysis

Statistical analyses were conducted using an unpaired Student’s *t*-test and one-way ANOVA multiple comparisons using GraphPad Prism 8 (GraphPad Software Inc, San Diego, CA, USA). Linear correlation analysis was also performed to evaluate the correlation between different RNA modifications or RNA modifications and RNA modification enzymes as determined by GraphPad Prism 8. Circos were drawn by R packages circlize, and the correlation between modification was calculated based on the method “Spearman.” Data were present as the mean ± standard error of mean (sem.), and *p* value < 0.05 was considered statistically significant. Each statistical test used for each figure is described in the legends. We independently repeated all the molecular biology experiments at least trice, and all attempts to reproduce the results were successful.

## Supplementary Information

**Additional file 1: Fig. S1.** Detection of RNA modifications in mouse multiple tissues RNA. (A) Experimental procedures for detecting and quantifying RNA modifications in mouse tissues RNA. (B) List of applied nucleobase standards. (C) The percentage of modified and unmodified four nucleotides (Adenine, Uracil, Cytosine and Guanine) in mouse mRNA and the relative proportion of detected RNA modifications across multiple tissues (The sum of all RNA modification was considered as 100 and the percentage was average of each modification across six tissues) (*n* = 2, Additional file 10). (D) The relative proportion of detected RNA modifications in mRNA across six tissues. (E) The heatmaps showed the relative expression levels of each RNA modification to average across six mouse tissues in mRNA. (F) PCA analysis of RNA modifications in mRNA across multiple tissues.

**Additional file 2: Fig. S2.** Multiple linear dependent correlations among different RNA modifications. (A and B) The linear correlations analysis between RNA modifications in tRNA-enriched fragments and 17-50 nt sncRNAs across multiple tissues (brain, liver, heart, spleen, lung and testis) (*n* = 3, Additional file 10). (C and D) The linear correlations of some specific RNA modifications in different RNA classes across multiple tissues. The linear regression analysis was done by GraphPad 8, the linear equations. *p < 0.05, **p < 0.01, ***p < 0.001, ****p < 0.0001.

**Additional file 3: Fig. S3.** Hypoxia response and altered signature of RNA modifications in mouse tissue total RNA by hypobaric hypoxia exposure. (A) Levels of RDW, red blood cell volume distribution width in peripheral blood (*n* = 8~10, Additional file 12). (B) The hypoxia response pathways of different tissues. (C) Comparison of RNA modifications in tissue total RNA between NC and Hypo groups (*n* = 3~5, Additional files 10 and 11). *p < 0.05, **p < 0.01, ***p < 0.001, ****p < 0.0001, ns. not significant. All results are shown as mean ± SEM.

**Additional file 4: Fig. S4.** Scatter plot comparison of transcriptome between normal control mouse tissues and hypoxia mouse tissues (*n* = 3).

**Additional file 5: Fig. S5.** Purification of the single endo-tRNA from mouse liver. (A) The codon usage frequency of individual tRNA in mouse liver nuclear coding genes. (B) Purification diagram of liver individual tRNA. (C) Confirmation of purified individual endo-tRNA by northern blot and denatured PAGE. L, Leu; S, Ser; E, Glu; A, Ala; G, Gly; P, Pro; V, Val; K, Lys; R, Arg; T, Thr; Q, Gln; D, Asp; I, Ile; F, Phe; N, Asn; H, His; Y, Tyr; C, Cys; M, Met; W, Trp; *, Stop codon; NB, northern blot; DP, denatured PAGE.

**Additional file 6: Fig. S6.** Scatter plot comparison of transcriptome between GC-2spd which transfected with NC group testis tRNA enriched fragments and hypoxia group testis tRNA enriched fragments.

**Additional file 7: Fig. S7.** Original uncropped images for liver single RNA. (A-E) The original uncropped images of northern blot to identify the pull-down products of tRNA^Ala^ (A), tRNA^Val^ (B), tRNA^Gly^ (C), tRNA^Leu^ (D) and tRNA^Glu^ (E). (F-I) The original uncropped images of denatured urea gel to identify the purified tRNA^Glu^ (F), tRNA^Gly^ (G), tRNA^Ala^ and tRNA^Val^ (H) and tRNA^Leu^.

**Additional file 8: Fig. S8.** Original uncropped images for tRNA enriched fragments stability analysis. (A-D) The original uncropped images of native gel to analyze liver tRNA-enriched fragments (A), testis tRNA enriched fragments stability (B), liver tRNA^Ala^ and liver tRNA^Gly^ (C), and liver tRNA^Val^ (D) stability against RNase A/T1 and fetal bovine serum.

**Additional file 9: Fig. S9.** Original uncropped images for denatured urea gel, northern blot and western blot. (A) Denatured urea gel image of liver total RNA for Fig. 6D. (B-D) Northern blot images of tRNA^Ala^ (B), tRNA^Val^ (C), tRNA^Gly^ (D) for Fig. 6D. (E) Denatured urea gel image of GC-2spd cell total RNA for Fig. 7D. (F-H) Northern blot images of tRNA^Ala^ for Fig. 7D. (I-K) Western blot images of Actin (I), eIF4A (J) and eIF4E (K) expressed in GC-2spd cells.

**Additional file 10: Table S1-S5.** Data for RNA modification levels in different RNA classes across normal control group mouse tissues. **Table S1.** Original data for RNA modification levels in total RNA of normal control mouse tissues. **Table S2.** Original data for RNA modification levels in tRNA-enriched RNA fragments of normal control mouse tissues. **Table S3.** Original data for RNA modification levels in 17-50 nt sncRNAs of normal control mouse tissues. **Table S4.** Original data for RNA modification levels in PolyA-enriched RNA of normal control mouse tissues. **Table S5.** Original data for RNA modification levels in liver single tRNA of normal control mouse tissues.

**Additional file 11: Table S6-S9.** Data for RNA modification levels in different RNA classes across hypoxia group mouse tissues. **Table S6.** Original data for RNA modification levels in total RNA of normal control and hypoxia mouse tissues. **Table S7.** Original data for RNA modification levels in tRNA-enriched RNA fragments of hypoxia mouse tissues. **Table S8.** Original data for RNA modification levels in 17-50 nt sncRNAs RNA of hypoxia mouse tissues. **Table S9.** Original data for RNA modification levels in liver single tRNA of normal control mouse tissues.

**Additional file 12: Table S10.** Data for routine analysis of blood.

**Additional file 13: Table S11-S12.** Data for GC-2spd growth analysis. **Table S11.** Fold change data for cell number of GC-2spd growth analysis. **Table S12.** CCK8 data for GC-2spd growth analysis.

## Data Availability

All data generated or analyzed during this study are included in this published article, its supplementary information files, and publicly available repositories. The raw data of transcriptome sequencing has been deposited into *figshare* website (DOI: 10.6084/m9.figshare.21981035). Data for RNA modification abundance obtained by LC-MS/MS analysis are available in Additional files 10 and 11. Data for routine analysis of blood are provided in Additional file 12. Data for GC-2spd cell growth analysis are provided in Additional file 13.

## Abbreviations

AMS: Acute mountain sickness
mRNA: Messenger RNA
rRNA: Ribosome RNA
tRNA: Transfer RNA
tsRNAs: tRNA-derived small noncoding RNAs
nt: Nucleotides
sncRNAs: Small noncoding RNAs
LC-MS/MS: Liquid chromatography-tandem mass spectroscopy
A: Adenine
U: Uracil
C: Cytosine
G: Guanine
RBC: Red blood cells
HGB: Hemoglobin concentration
MCH: Mean corpuscular hemoglobin
WBC: White blood cell
RNA-Seq: RNA sequencing
FBS: Fetal bovine serum
GC-2spd: Germ cell-2 spermatid
SNR: Signal-to-noise ratio
EPO: Erythropoietin
HIF-1: Hypoxia-inducible factor-1
HSC: Hematopoietic stem cell
