# Supplementary figures and images for: Hypoxia induces alterations in tRNA modifications involved in translational control
Source: BMC Biol. 2023 Feb 21;21:39. doi: 10.1186/s12915-023-01537-x (PMC9942361; doi:10.1186/s12915-023-01537-x)

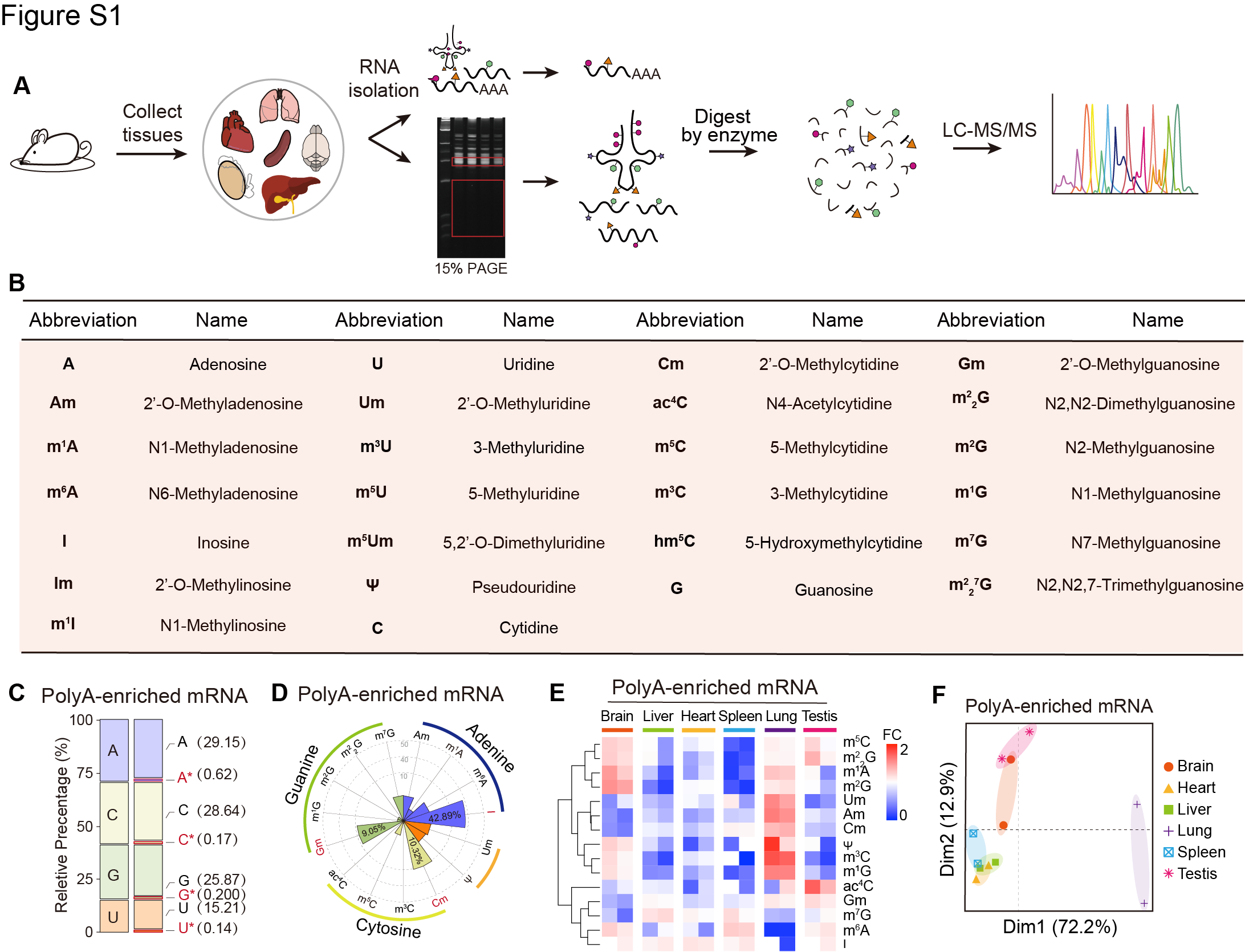

Supplement: Supplementary file 1 — Additional file 1: Fig. S1. Detection of RNA modifications in mouse multiple tissues RNA. (A) Experimental procedures for detecting and quantifying RNA modifications in mouse tissues RNA. (B) List of applied nucleobase standards. (C) The percentage of modified and unmodified four nucleotides (Adenine, Uracil, Cytosine and Guanine) in mouse mRNA and the relative proportion of detected RNA modifications across multiple tissues (The sum of all RNA modification was considered as 100 and the percentage was average of each modification across six tissues) (n = 2, Additional file 10). (D) The relative proportion of detected RNA modifications in mRNA across six tissues. (E) The heatmaps showed the relative expression levels of each RNA modification to average across six mouse tissues in mRNA. (F) PCA analysis of RNA modifications in mRNA across multiple tissues. [file 12915_2023_1537_MOESM1_ESM.tif]

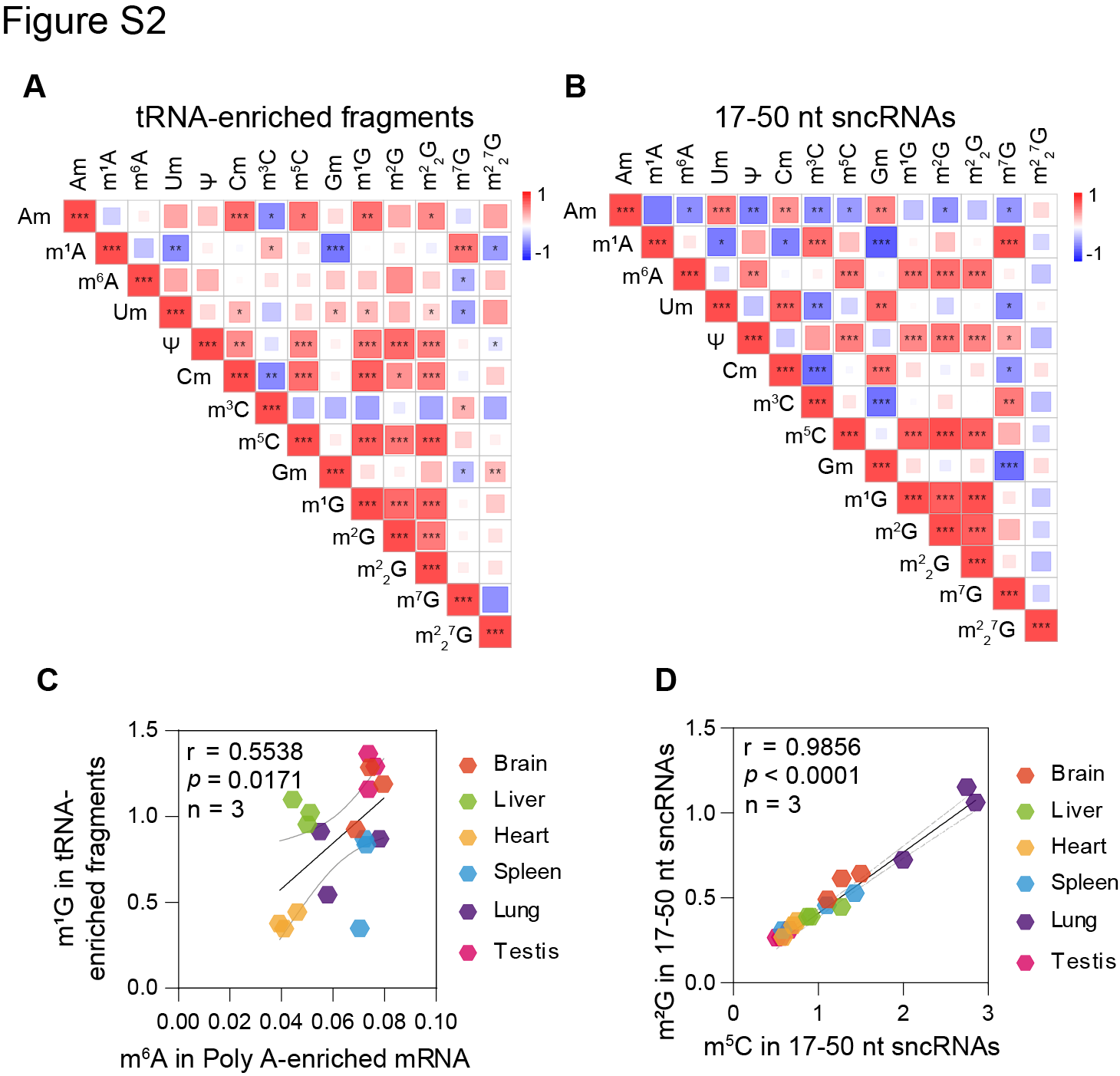

Supplement: Supplementary file 2 — Additional file 2: Fig. S2. Multiple linear dependent correlations among different RNA modifications. (A and B) The linear correlations analysis between RNA modifications in tRNA-enriched fragments and 17-50 nt sncRNAs across multiple tissues (brain, liver, heart, spleen, lung and testis) (n = 3, Additional file 10). (C and D) The linear correlations of some specific RNA modifications in different RNA classes across multiple tissues. The linear regression analysis was done by GraphPad 8, the linear equations. *p < 0.05, **p < 0.01, ***p < 0.001, ****p < 0.0001. [file 12915_2023_1537_MOESM2_ESM.tif]

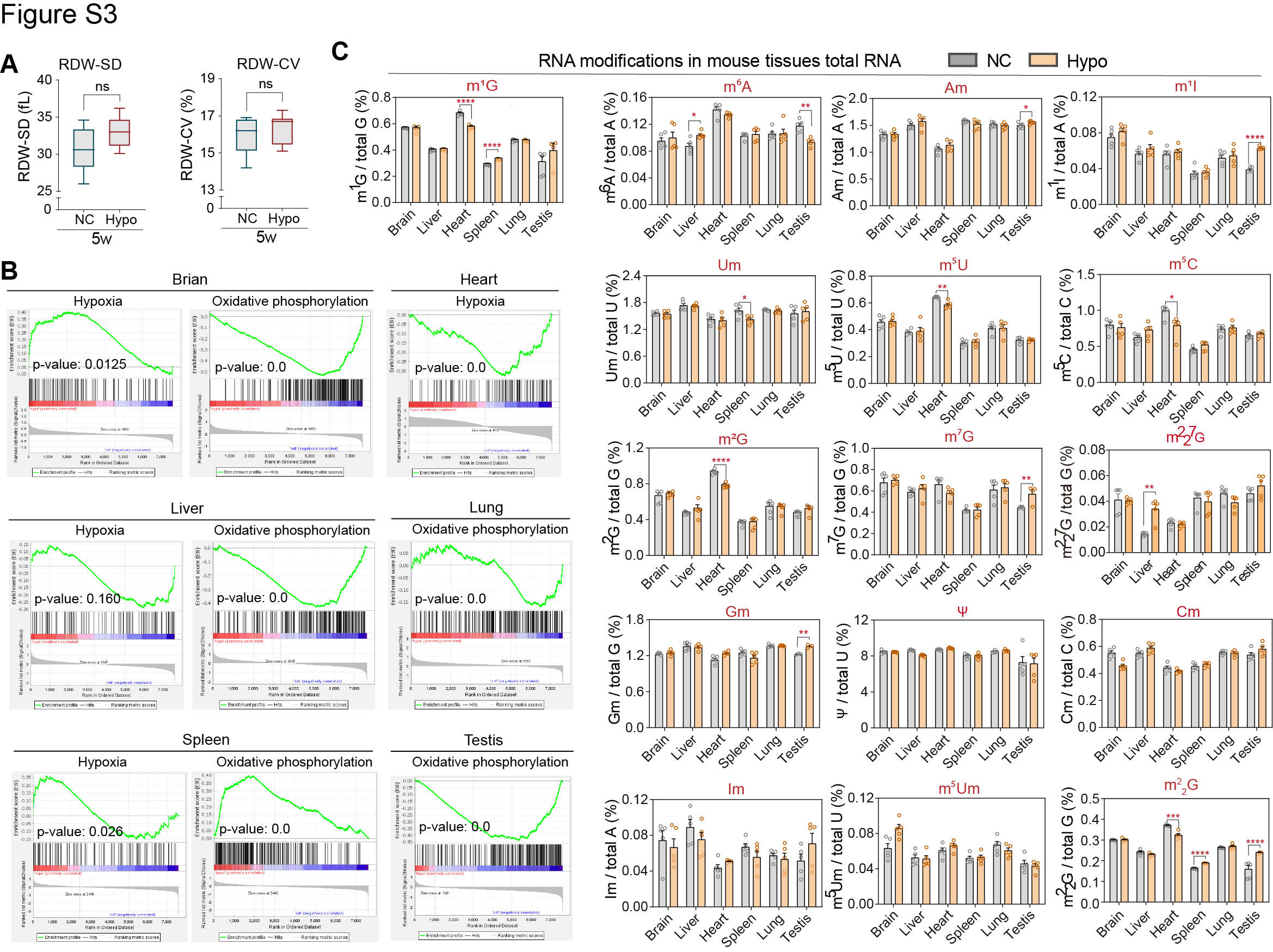

Supplement: Supplementary file 3 — Additional file 3: Fig. S3. Hypoxia response and altered signature of RNA modifications in mouse tissue total RNA by hypobaric hypoxia exposure. (A) Levels of RDW, red blood cell volume distribution width in peripheral blood (n = 8~10, Additional file 12). (B) The hypoxia response pathways of different tissues. (C) Comparison of RNA modifications in tissue total RNA between NC and Hypo groups (n = 3~5, Additional files 10 and 11). *p < 0.05, **p < 0.01, ***p < 0.001, ****p < 0.0001, ns. not significant. All results are shown as mean ± SEM. [file 12915_2023_1537_MOESM3_ESM.tif]

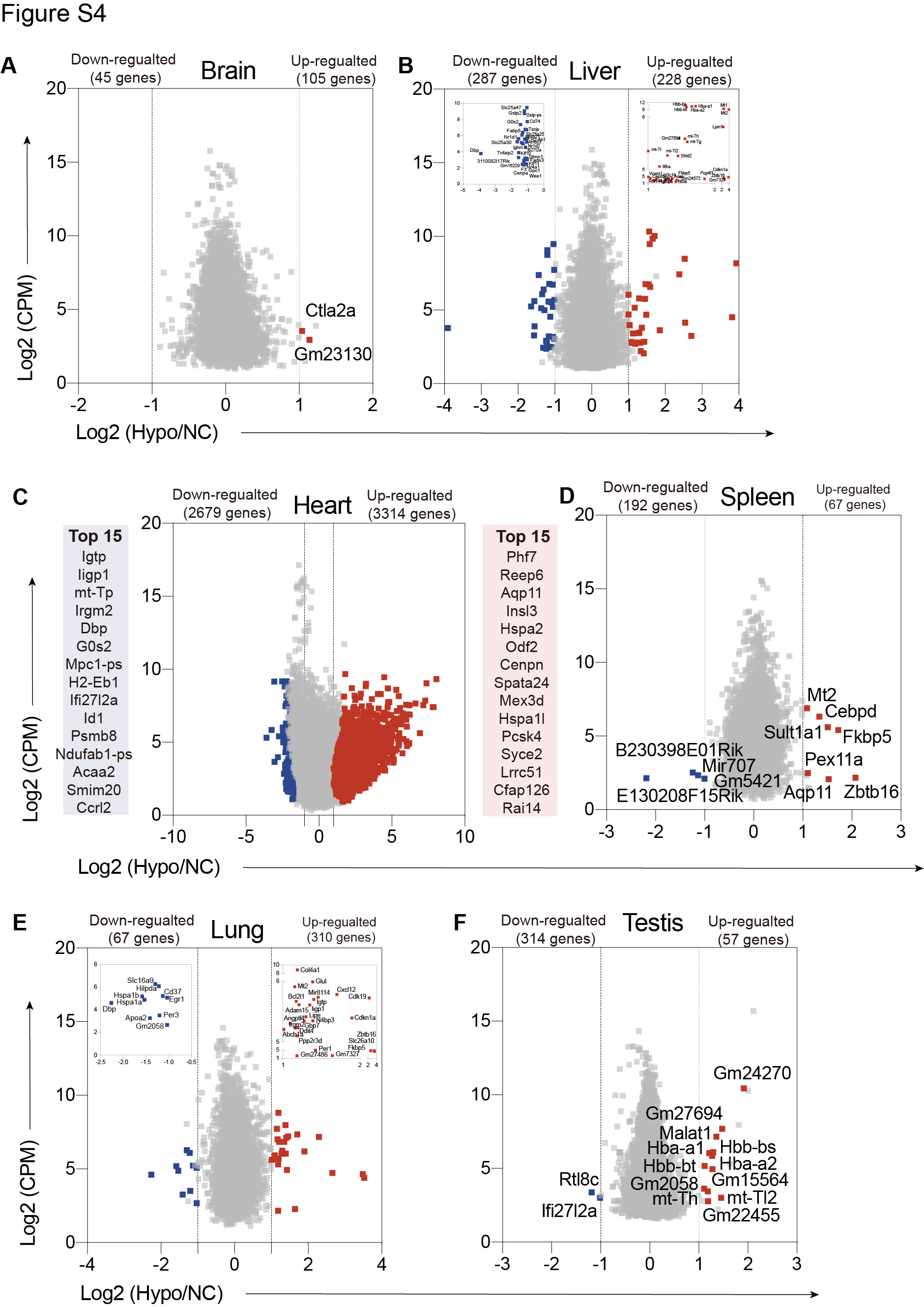

Supplement: Supplementary file 4 — Additional file 4: Fig. S4. Scatter plot comparison of transcriptome between normal control mouse tissues and hypoxia mouse tissues (n = 3). [file 12915_2023_1537_MOESM4_ESM.tif]

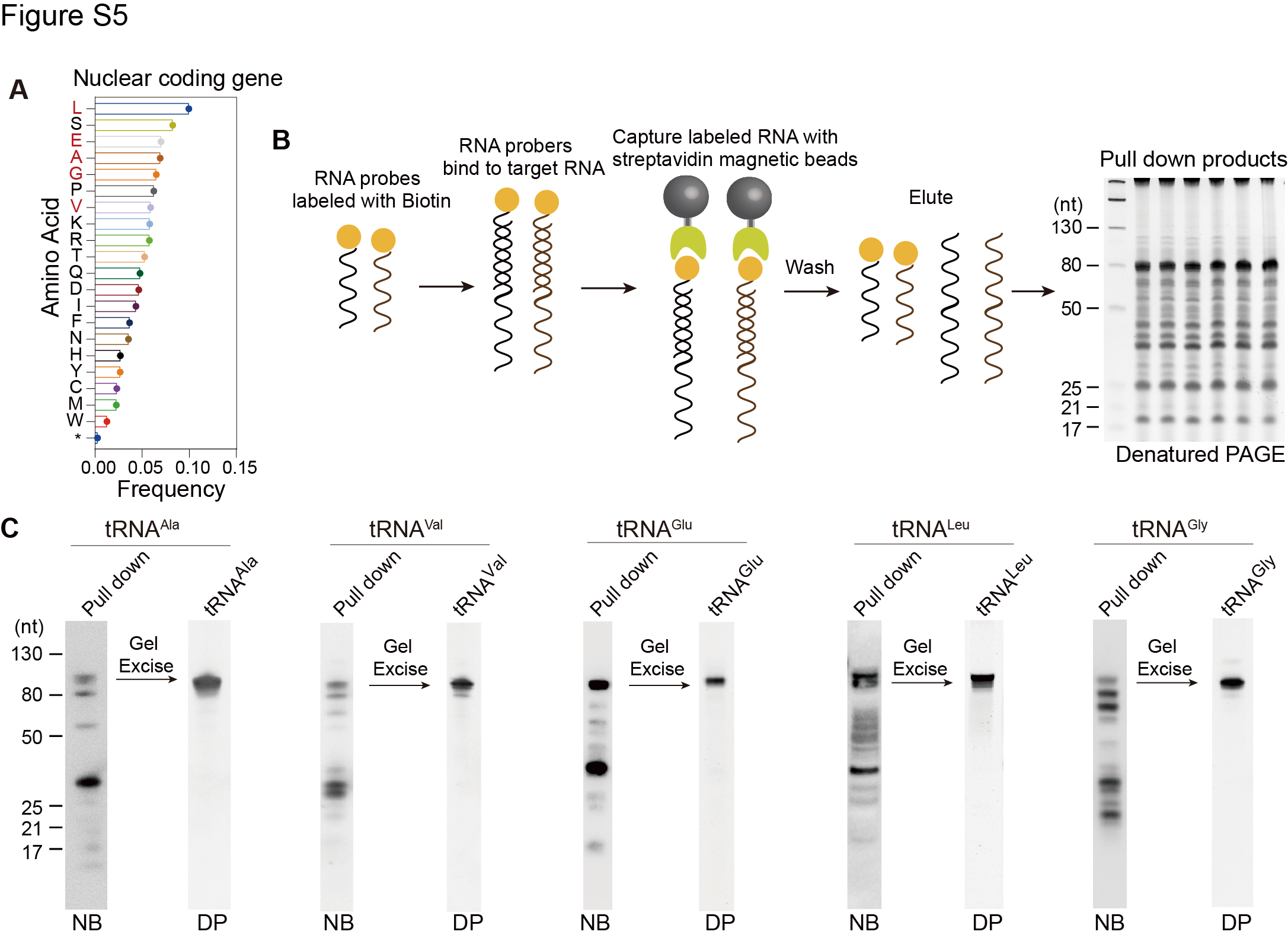

Supplement: Supplementary file 5 — Additional file 5: Fig. S5. Purification of the single endo-tRNA from mouse liver. (A) The codon usage frequency of individual tRNA in mouse liver nuclear coding genes. (B) Purification diagram of liver individual tRNA. (C) Confirmation of purified individual endo-tRNA by northern blot and denatured PAGE. L, Leu; S, Ser; E, Glu; A, Ala; G, Gly; P, Pro; V, Val; K, Lys; R, Arg; T, Thr; Q, Gln; D, Asp; I, Ile; F, Phe; N, Asn; H, His; Y, Tyr; C, Cys; M, Met; W, Trp; *, Stop codon; NB, northern blot; DP, denatured PAGE. [file 12915_2023_1537_MOESM5_ESM.tif]

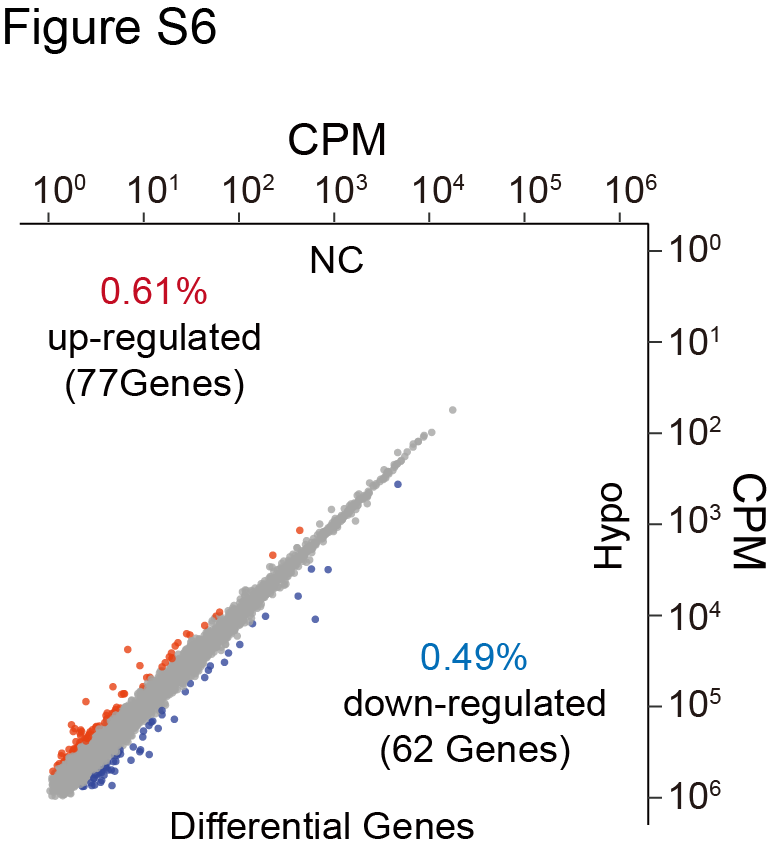

Supplement: Supplementary file 6 — Additional file 6: Fig. S6. Scatter plot comparison of transcriptome between GC-2spd which transfected with NC group testis tRNA enriched fragments and hypoxia group testis tRNA enriched fragments. [file 12915_2023_1537_MOESM6_ESM.tif]

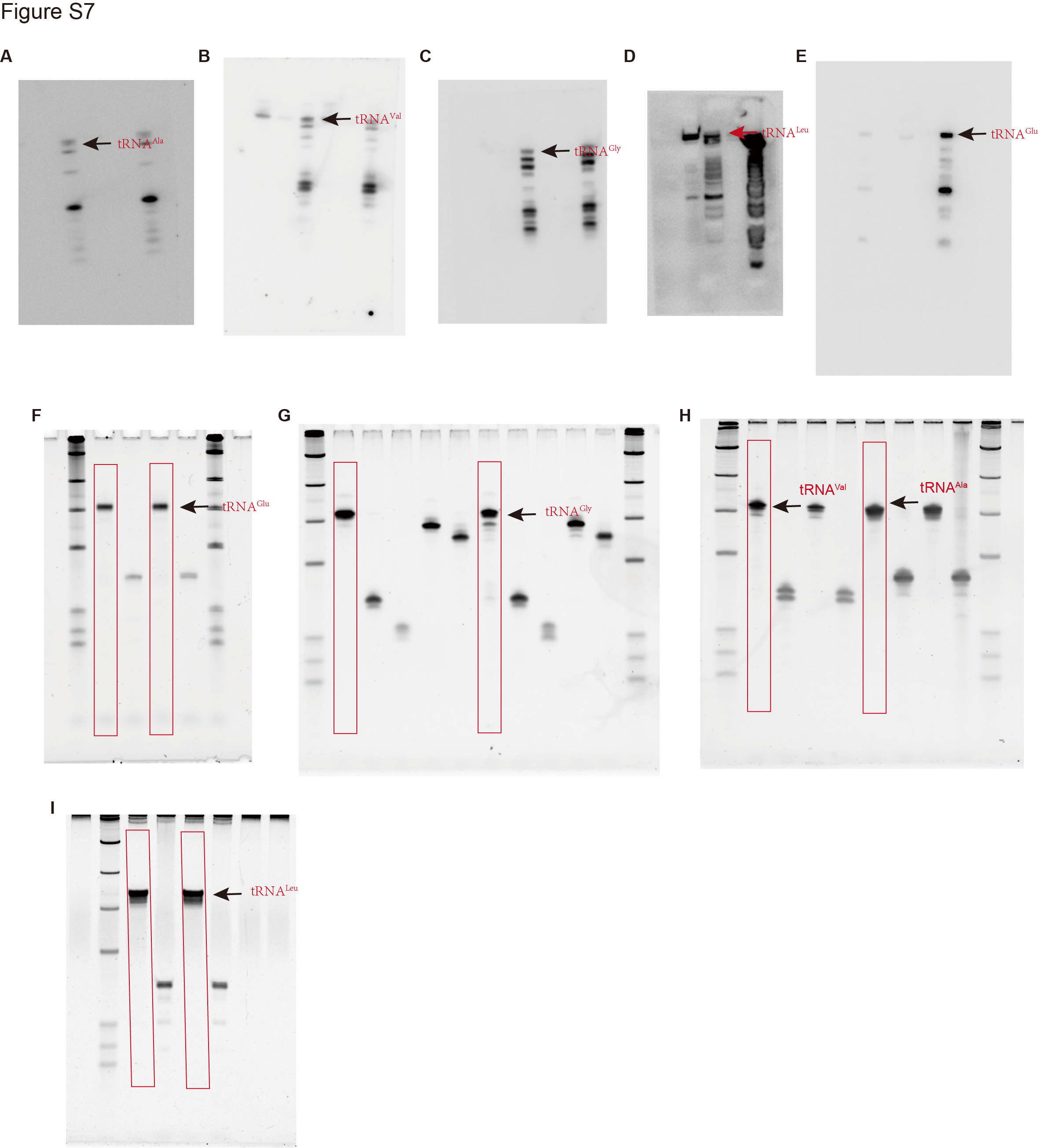

Supplement: Supplementary file 7 — Additional file 7: Fig. S7. Original uncropped images for liver single RNA. (A-E) The original uncropped images of northern blot to identify the pull-down products of tRNAAla (A), tRNAVal (B), tRNAGly (C), tRNALeu (D) and tRNAGlu (E). (F-I) The original uncropped images of denatured urea gel to identify the purified tRNAGlu (F), tRNAGly (G), tRNAAla and tRNAVal (H) and tRNALeu. [file 12915_2023_1537_MOESM7_ESM.tif]

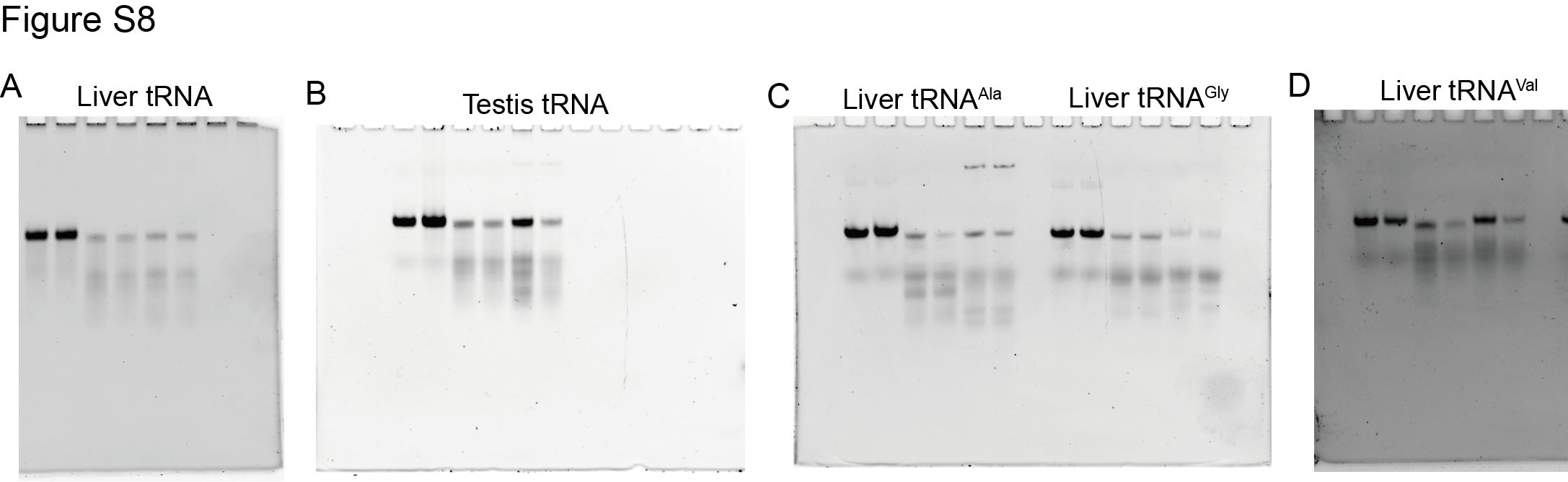

Supplement: Supplementary file 8 — Additional file 8: Fig. S8. Original uncropped images for tRNA enriched fragments stability analysis. (A-D) The original uncropped images of native gel to analyze liver tRNA-enriched fragments (A), testis tRNA enriched fragments stability (B), liver tRNAAla and liver tRNAGly (C), and liver tRNAVal (D) stability against RNase A/T1 and fetal bovine serum. [file 12915_2023_1537_MOESM8_ESM.tif]

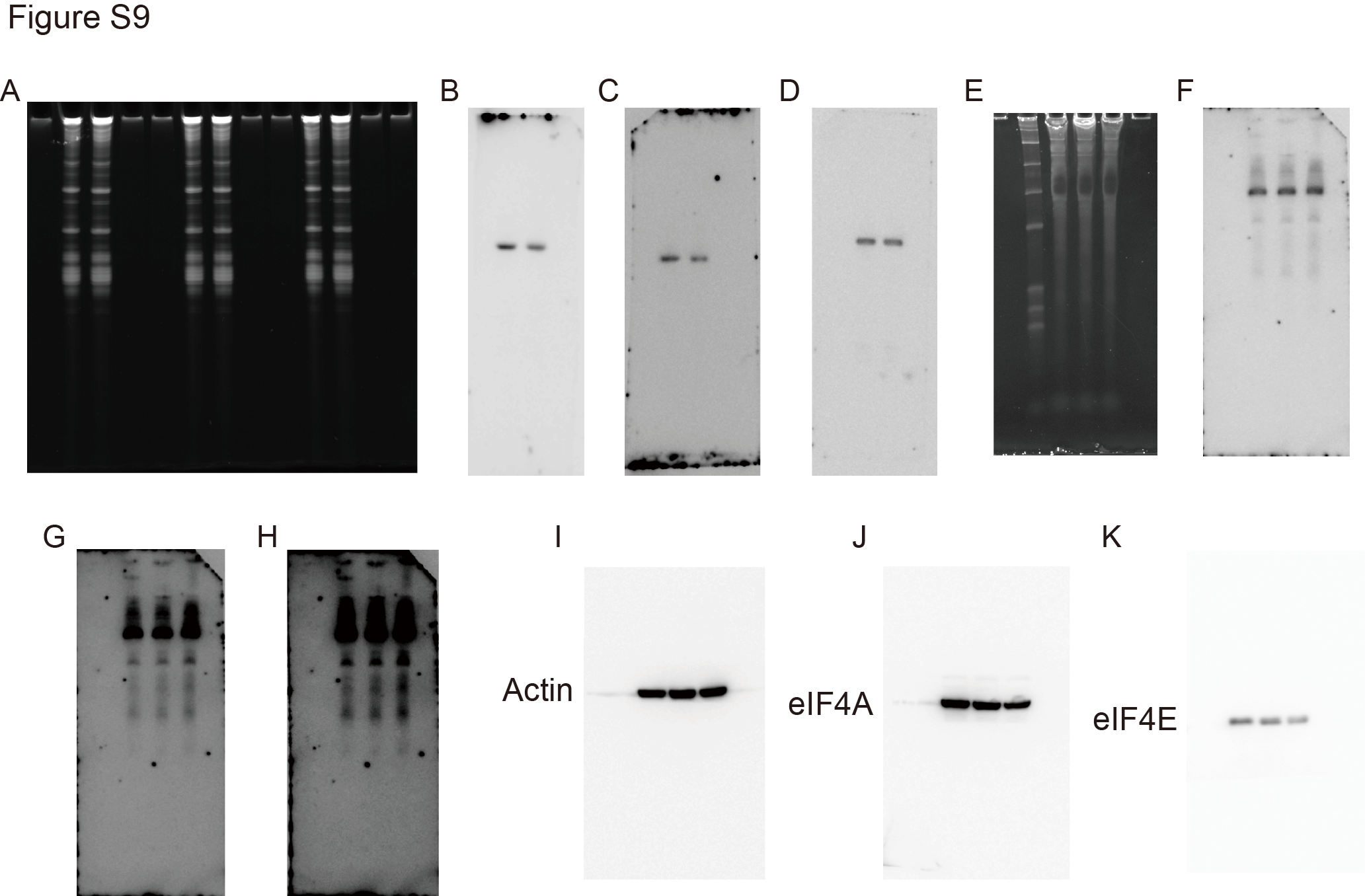

Supplement: Supplementary file 9 — Additional file 9: Fig. S9. Original uncropped images for denatured urea gel, northern blot and western blot. (A) Denatured urea gel image of liver total RNA for Fig. 6D. (B-D) Northern blot images of tRNAAla (B), tRNAVal (C), tRNAGly (D) for Fig. 6D. (E) Denatured urea gel image of GC-2spd cell total RNA for Fig. 7D. (F-H) Northern blot images of tRNAAla for Fig. 7D. (I-K) Western blot images of Actin (I), eIF4A (J) and eIF4E (K) expressed in GC-2spd cells. [file 12915_2023_1537_MOESM9_ESM.tif]
